# Systematic Analysis of the Global, Regional and National Burden of Cardiovascular Diseases from 1990 to 2017

**DOI:** 10.1007/s44197-021-00024-2

**Published:** 2021-12-13

**Authors:** Zhenkun Wang, Aihua Du, Hong Liu, Ziwei Wang, Jifa Hu

**Affiliations:** 1 Department of Scientific Research, Tongji Hospital, Tongji Medical College, Huazhong University of Science and Technology, Wuhan, 430030 China; 2 The Central Hospital of Wuhan, Tongji Medical College, Huazhong University of Science and Technology, Wuhan, 430014 China

**Keywords:** Cardiovascular diseases (CVDs), Mortality, Disability-adjusted life years (DALYs), Risk factors, Global burden of diseases (GBD)

## Abstract

**Background:**

Previous studies on the burden of cardiovascular diseases (CVDs) were mainly based on limited data of the study period or area, or did not include detailed risk factor analysis.

**Objective:**

To investigate up-to-date temporal and regional trends and risk factors of mortality and disability-adjusted life years (DALYs) attributed to CVDs by age, sex, and disease throughout the world.

**Methods:**

Data for the disease burden of CVDs in 195 countries and territories from 1990 to 2017, including mortality, DALYs, age-standardized mortality rates, and age-standardized DALY rates, were estimated from the Global Burden of Disease Study 2017. Risk factors attributable to deaths and DALYs for CVDs were also estimated using the comparative risk assessment framework.

**Results:**

The number of deaths from CVDs increased by 48.62%, from 11.94 (95% UI 11.78–12.18) million in 1990 to 17.79 (17.53–18.04) million in 2017. However, the age-standardized mortality rate decreased by an average of − 1.45% (− 1.72% to − 1.18%) annually. After fluctuation in the expected age-standardized mortality rate of CVDs in most of the socio-demographic index (SDI) scale, these rates decrease rapidly for SDI values of 0.7 and higher. In 2017, metabolic risks accounted for 73.48% of deaths and 73.25% of DALYs due to CVDs, behavioral factors accounted for 63.23% of deaths and 66.71% of attributable DALYs.

**Conclusion:**

CVDs remain a major global health burden due to the increment in death numbers and DALYs. Aging and the main risk factors are the main drivers of mortality and health loss. More attention to main risk factors should be paid with supportive health policies.

**Supplementary Information:**

The online version contains supplementary material available at 10.1007/s44197-021-00024-2.

## Introduction

Cardiovascular diseases (CVDs) are a serious public health burden, with approximately 17.79 million deaths in 2017, consisting of 31.80% of all deaths worldwide. Ischemic heart disease and stroke are the foremost common types of CVDs, whereas hypertensive heart disease is also a pressing global health issue. These three types of CVDs are mainly related to demographic patterns, socio-economic development, and potentially related risk factors, including metabolic risks, dietary risks, tobacco, et al. In the past several decades, the elderly population has grown quickly [1]. Exposure to metabolic risks has been increasing to somehow offset health promotion from the improvement of environmental and behavioral risks for non-communicable diseases (NCDs) [2]. Meanwhile, socio-economic development, population structure, and various risk factors have changed substantially during the same period [3].

Previous studies on mortality and health burden from CVDs were mainly based on limited data of the study period or area, or did not include detailed risk factor analysis [4–7]. This study provides a systematic view of the temporal and regional trends of mortality and disability-adjusted life years (DALYs) attributed to CVDs, by age and sex, throughout the world from 1990 to 2017, utilizing data from the Global Burden of Disease Study 2017 (GBD 2017). The main influencing factors of CVDs, including the socio-demographic index and various risk exposures, were analyzed to assist governments and relevant policymakers in lessening mortality from CVDs, and promoting health and quality of life.

## Methods

### Data Source

Data for the disease burden of CVDs in 195 countries and territories from 1990 to 2017, including mortality, DALYs, age-standardized mortality rates, and age-standardized DALY rates, were obtained from the GBD 2017 [8, 9]. The International Classification of Diseases, Tenth Revision (ICD-10) was used to identify the cases of CVDs, including ischemic heart disease, stroke, and hypertensive heart disease (supplementary Table 1) [8]. The quality and comparability of the data were evaluated and improved through a series of means in GBD 2017 as reported previously. Mortality was estimated using vital registration data coded to the ICD system or verbal autopsy.

The main influencing factors for CVDs included the socio-demographic index (SDI) and the related risk factors. The SDI is the geometric mean of 0–1 index of per-capita income, mean education for those ages 15 and older, and total fertility rate under the age of 25 [8]. As a composite, a location with an SDI of 0 would have a theoretical minimum level of development relevant to health, while a location with an SDI of 1 would have a theoretical maximum level. The methods utilized to compute the index are described in the previous study [9]. The SDI value was used to categorize the countries and territories into five SDI quintiles (high, high–middle, middle, low–middle, and low levels). The cut-off values adopted to define the quintile for analysis were calculated using country/territory level estimates of the SDI for the year 2017, excluding those with populations of less than one million [9].

The GBD 2017 study incorporated the comparative risk assessment framework to define risk exposures, including environmental and occupational risks (air pollution, lead exposure, etc.), behavioral risks (dietary risks, tobacco, etc.), and metabolic risks (high body mass index, high fasting plasma glucose, high LDL cholesterol, high systolic blood pressure, etc.), and to quantify the corresponding burden of them. The risk factor hierarchy information and relevant definitions of risk factors were described in detail in the previous study [2].

### Statistical Analyses

The standardized methods of the GBD 2017 have been extensively reported [8, 9]. Mortality and DALYs from CVDs—ischemic heart disease, stroke, and hypertensive heart disease—were estimated for 195 countries and territories, by age and sex, from 1990 to 2017 using DisMod-MR 2, which exploits the fact that disease incidence, prevalence, remission, case fatality, and mortality are not independent variables [8, 9]. To define uncertainty of each variable for all related data in this study, 95% uncertainty intervals (UI) were calculated. All entities quantified in GBD 2017 were generated from the mean of 1000 draws, and 95% UIs were determined using the 2.5th and 97.5th percentiles of the ordered 1000 draws. The estimated annual percentage change (EAPC) is a general and extensively adopted indicator of the age-standardized rate trend over a specified interval [10, 11]. The EAPC of the age-standardized mortality rate was generated by means of a generalized linear model with a Poisson distribution [12]. All statistical analyses were performed using the R program (Version 3.6.2, R core team). A p value of less than 0.05 was considered statistically significant.

### Patient and Public Involvement

There has been no patient and/or public involvement in the study design, data collection, data analysis, and writing of this research.

## Results

### Disease Burden and Mortality Estimates

DALYs due to CVDs increased from 266.82 to 365.87 million during 1990–2017. In Table 1, deterioration in DALYs was seen in patients with ischemic heart disease, stroke, and hypertensive heart disease. The number of deaths from CVDs increased by 48.62%, from 11.94 (95% UI 11.78–12.18) million in 1990 to 17.79 (17.53–18.04) million in 2017. However, the age-standardized mortality rate decreased from 334.70 (330.50–341.57) per 100,000 people to 233.07 (229.66–236.38) per 100,000 people, by an average of − 1.45% (− 1.72% to − 1.18%) annually during the same period, and which varied to a large extent between males and females both in the year 1990 and 2017.

**Table 1:** The global deaths, ASMR, and DALY due to cardiovascular diseases in 1990 and 2017

Year	Measure	Cardiovascular diseases	Ischemic heart disease	Stroke	Hypertensive heart disease
1990	DALY (10^6^)	266.82 (260.27–273.59)*	119.48 (116.95–122.55)	98.88 (95.63–102.51)	11.08 (8.80–12.01)
	No. of deaths (10^3^)	11941.53 (11784.72–12179.15)	5865.21 (5771.95–5999.77)	4362.50 (4272.31–4519.81)	539.87 (424.81–586.34)
	ASMR (/10^5^)				
	Both	334.70 (330.50–341.57)	166.97 (164.52–170.76)	120.80 (118.40–125.26)	15.22 (11.94–16.52)
	Male	379.38 (372.47–386.86)	199.94 (195.70–205.72)	130.97 (127.37–135.59)	14.40 (10.49–15.55)
	Female	297.38 (292.42–305.56)	139.66 (137.43–143.51)	112.30 (109.54–117.18)	15.69 (12.35–17.31)
2017	DALY (10^6^)	365.87 (355.16–376.75)	170.28 (167.14–174.05)	132.05 (126.50–137.35)	16.54 (12.74–17.88)
	No. of deaths (10^3^)	17,790.95 (17527.08–18042.67)	8930.37 (8790.70–9138.68)	6167.29 (6044.26–6327.60)	925.68 (681.44–994.94)
	ASMR (/10^5^)				
	Both	233.07 (229.66–236.38)	116.95 (115.13–119.71)	80.45 (78.86–82.56)	12.27 (8.98–13.20)
	Male	275.50 (270.45–280.56)	144.37 (141.53–147.89)	92.95 (90.40–95.58)	12.28 (8.22–13.25)
	Female	196.11 (191.95–200.13)	93.32 (91.18–96.05)	69.60 (67.73–71.89)	12.13 (8.62–13.24)

*DALY*, disability-adjusted life year; *ASMR*, age-standardized mortality rate

*Data in parentheses shown as 95% confidence interval

**Fig. 1:**
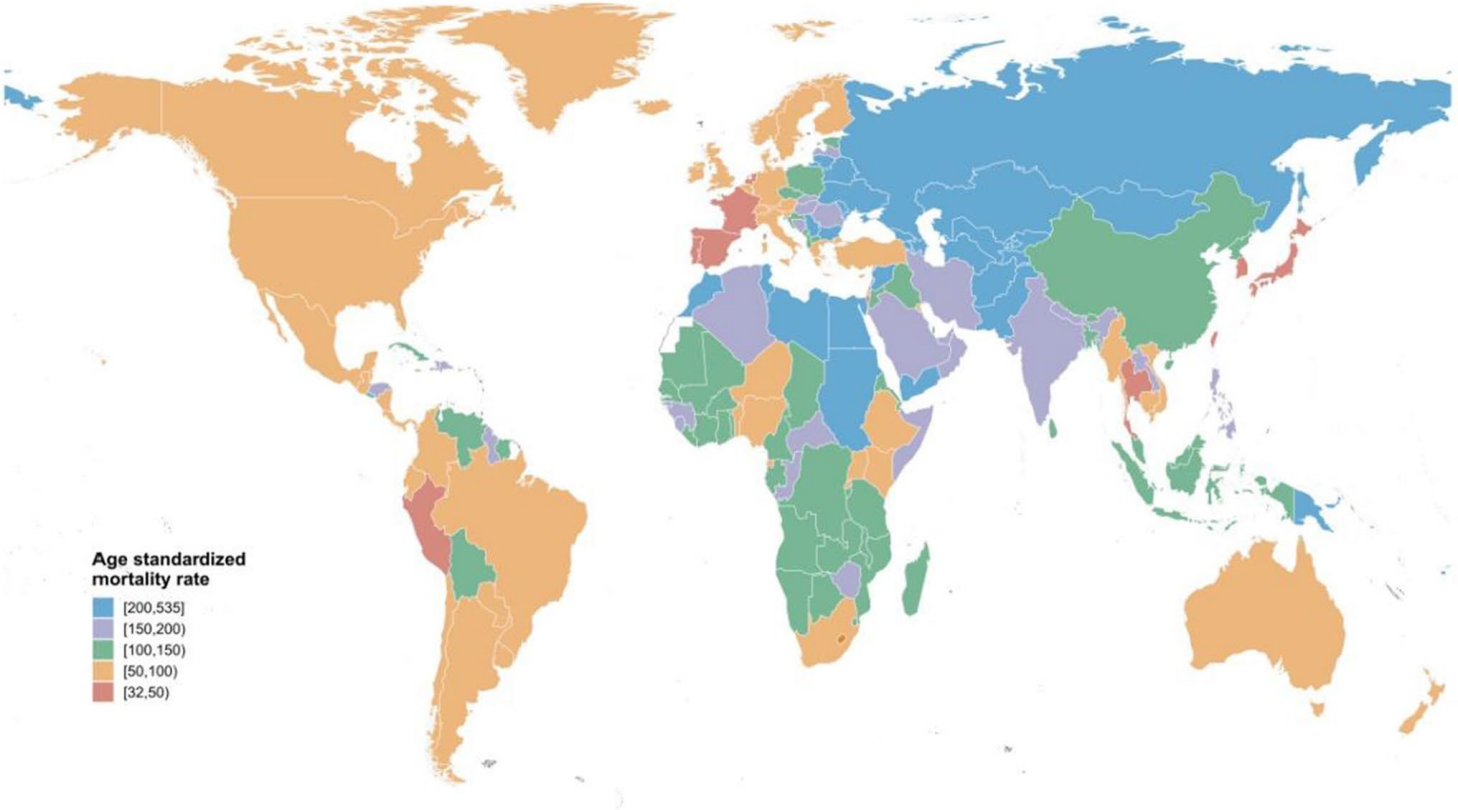
Global age-standardized mortality rate per 100,000 people of ischemic heart disease for both sexes combined in 195 countries and territories in 2017

**Fig. 2:**
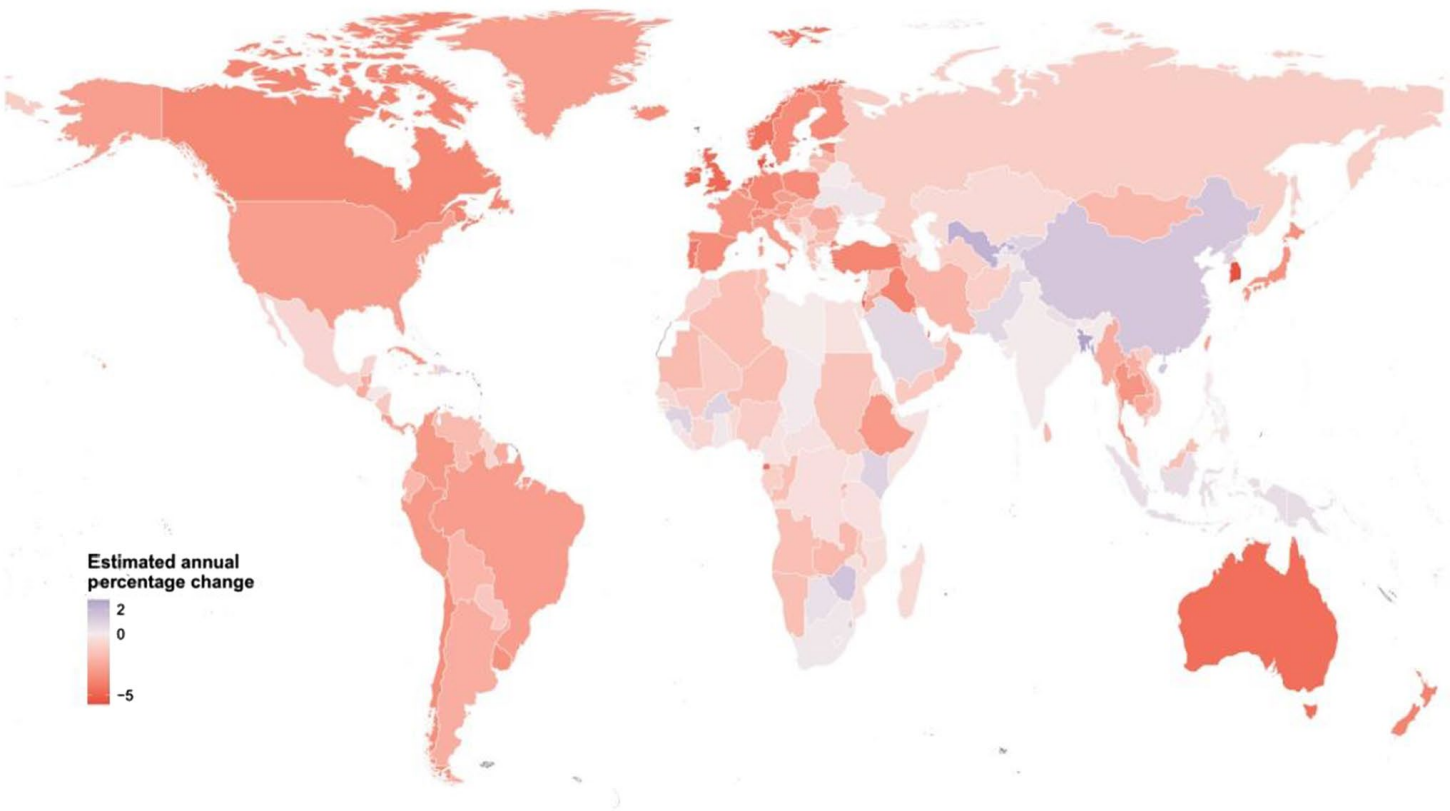
Estimated annual percentage change in age-standardized mortality rate per 100,000 people of ischemic heart disease for both sexes combined in 195 countries and territories from 1990 to 2017

In 2017, there were 8.93 (95% UI 8.79–9.14) million deaths due to ischemic heart disease, accounting for 50.20% of the total number of deaths from CVDs. Figure 1 illustrates a global perspective of a large range of age-standardized mortality rates attributable to ischemic heart disease in 2017. Estimates of mortality rates for each country/territory were computed and shown in supplementary table 2. During the period of 1990–2017, the global age-standardized mortality rate of ischemic heart disease dropped by an average of − 1.40% (− 1.78% to − 1.02%) annually. Figure 2 demonstrates that the mortality rates in 19 countries increased, and the mortality rate in Bangladesh increased at the fastest average rate of 2.79% (2.31–3.27%) annually; China, as the country with the largest number of deaths attributed to ischemic heart disease during 1990–2017, increased at an average rate of 1.39% (0.92–1.87%) annually.

In 2017, 6.17 (95% UI 6.04–6.33) million people died from stroke, accounting for 34.67% of the total number of deaths from CVDs. The age-standardized mortality rate of stroke differed considerably among countries (supplementary Fig. 1). The age-standardized mortality rate declined from 120.80 (118.40–125.26) per 100,000 people to 80.45 (78.86–82.56) per 100,000 people worldwide, with an average decrease of − 1.66% (− 2.11% to − 1.20%) annually from 1990 to 2017 (supplementary Fig. 2). The mortality rates in 10 countries rose, and the biggest rise annually was seen in the Philippines [1.74% (1.29–2.19)].

Supplementary Fig. 3 displays the global age-standardized mortality rate of hypertensive heart disease that varied widely in the regional distribution in 2017. An average decrease of − 0.86% (95% UI − 2.13% to 0.42%) annually in the global age-standardized mortality rate associated with hypertensive heart disease from 1990 to 2017 was seen (supplementary Fig. 4).

### Sex and Age Differences in Mortality and DALY

**Fig. 3:**
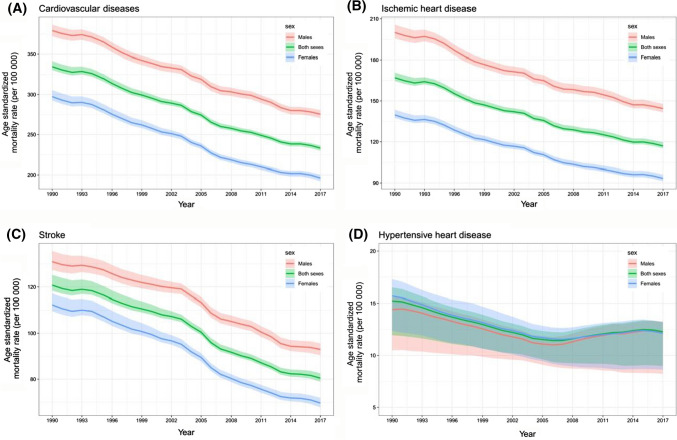
Age-standardized mortality rate per 100,000 people of cardiovascular diseases; ischemic heart disease; stroke; and hypertensive heart disease in males, females, and both sexes from 1990 to 2017. Shading indicates 95% uncertainty intervals

Continued declines were seen in the age-standardized DALY rates and age-standardized mortality rates of CVDs, ischemic heart disease, and stroke for both sexes from 1990 to 2017; whereas fluctuations after slight decrease were shown in the age-standardized DALY rates and age-standardized mortality rates of hypertensive heart disease (Fig. 3; supplementary Fig. 5). The age-standardized mortality rates of CVDs, ischemic heart disease, and stroke in males were higher than in females in this period. For hypertensive heart disease, the uncertainty intervals of mortality rate between males and females were almost overlapped. For hypertensive heart disease, the uncertainty intervals of mortality rate between males and females were almost overlapped during 1990–2017.

**Fig. 4:**
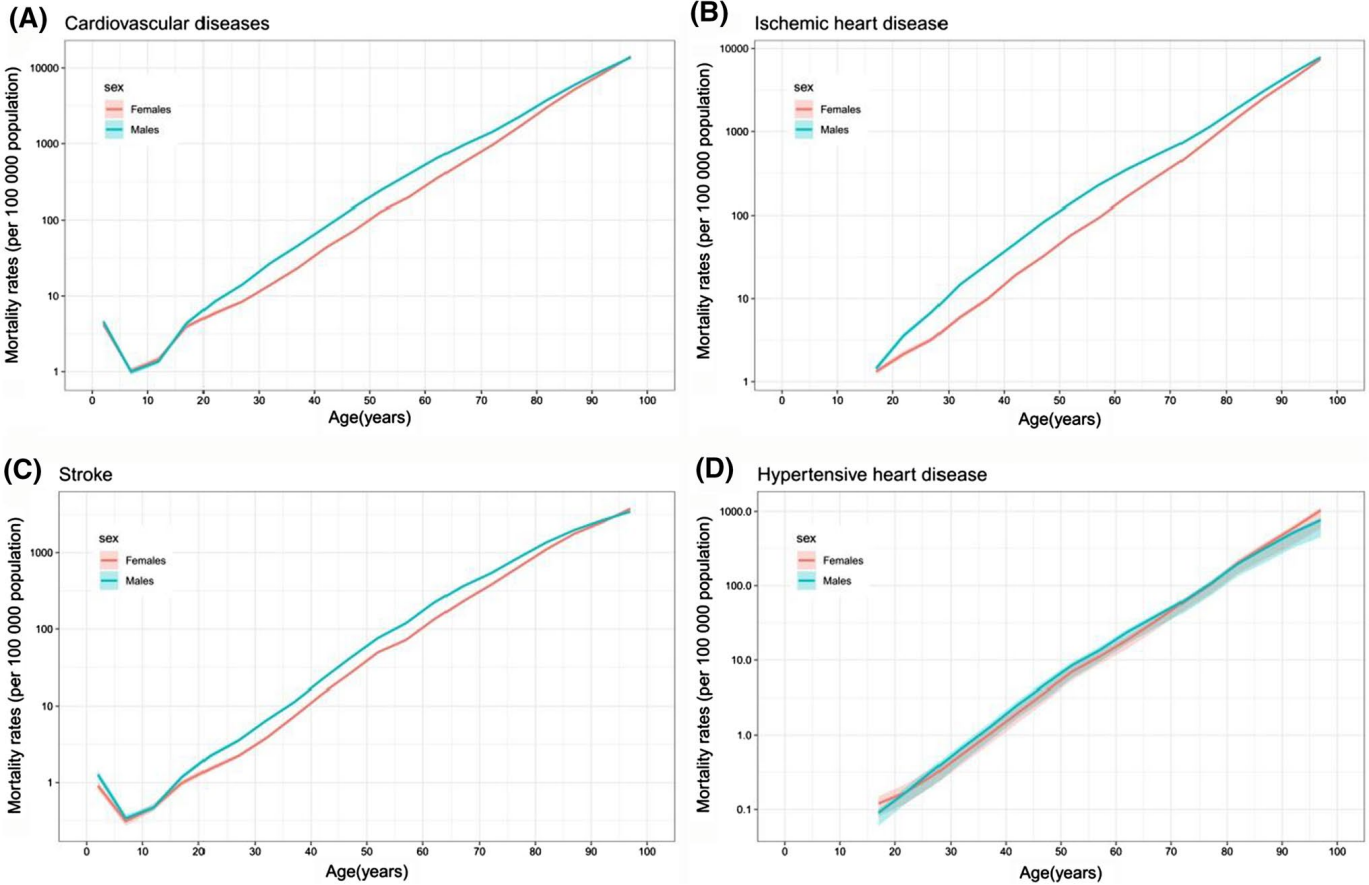
Global mortality rates per 100,000 people of cardiovascular diseases; ischemic heart disease; stroke; and hypertensive heart disease by age in males and females in 2017. The y axis is represented on a logarithmic scale. Shading indicates 95% uncertainty intervals (some of them were too narrow to show in the figures)

Deaths attributed globally to CVDs, ischemic heart disease, stroke, and hypertensive heart disease in 1990 and 2017 by age and sex are demonstrated in supplementary Figs. 6 and 4, respectively. The global mortality rates of the overall and all three types of CVDs increased exponentially with age among those aged 15 and older. Generally, the mortality rates of CVDs, ischemic heart disease, and stroke in males were higher than in females in most age groups, except that those mortality rates converged between males and females in the oldest and the youngest age groups. For hypertensive heart disease, the uncertainty intervals of mortality rate between males and females were largely overlapped in all age groups. For hypertensive heart disease, the uncertainty intervals of mortality rate between males and females were largely overlapped in all age groups (Fig. 4).

### Relation Between the Socio-Demographic Index and Estimates of Mortality and DALYs

**Fig. 5:**
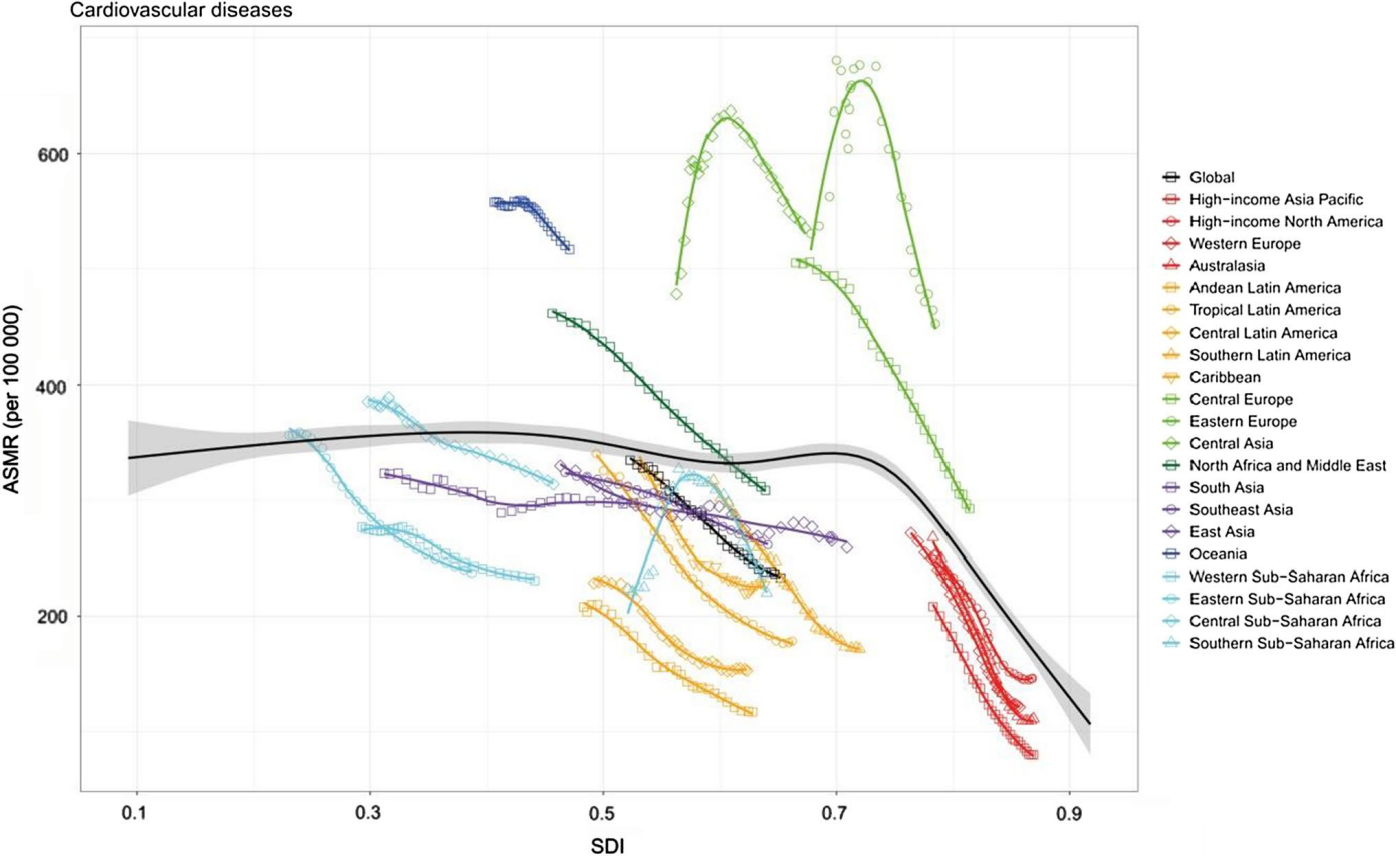
Age-standardized mortality rate of cardiovascular diseases for 21 GBD regions, 1990–2017. The longest black line shows expected values across the spectrum of the SDI. Shading indicates 95% confidence intervals

After fluctuation in the expected age-standardized mortality rate of CVDs in most parts of the SDI scale, these rates decrease rapidly for SDI values of 0.7 and higher (Fig. 5). For most regions, the mortality rates of CVDs decreased with increases in SDI over time. However, Eastern Europe and Central Asia saw increased mortality rates in the 1990s, followed by subsequent declines as SDI value rose. The mortality rates of CVDs in southern Sub-Saharan Africa showed a similar pattern, with an initial spike in rates with increasing SDI, followed by a steady decrease. The age-standardized mortality rates were higher than expected early in the time series for Eastern and Central Sub-Saharan Africa and in the period before 2010 for North Africa and the Middle East, but decreased with increasing SDI. Eastern Europe, Central Europe, Central Asia, and Oceania had higher CVDs-related mortality rates than would be expected based on comparisons of SDI for all years. Conversely, the other remaining regions were lower than the corresponding values for all years. For the three types of CVDs, although the direction and magnitude of the fluctuations were not consistent in most parts of the SDI scale, the mortality rates all decreased rapidly for SDI values of 0.7 and higher (supplementary Figs. 7, 8, and 9). Similarly, the relation between the socio-demographic index and age-standardized DALY rates resembled those between the socio-demographic index and mortality rates of the overall and the three types of CVDs.

### Risk Factors

According to the comparative risk assessment framework, most deaths [85.94% (95% UI 84.05–87.65)] and DALYs [85.82% (84.51–87.00)] due to CVDs can be attributed to risk factors measured in the GBD 2017. The remaining burden is due to unknown or unmeasured risk factors, genetic factors, or gene–environment interactions. Metabolic risks accounted for 73.48% (68.40–78.12) of deaths and 73.25% (68.98–77.21) of DALYs due to CVDs. Behavioral factors (dietary risks, tobacco, low physical activity, and alcohol use) accounted for 63.23% (59.93–66.44) of deaths and 66.71% (63.69–69.28) of attributable DALYs, and environmental risks (air pollution and lead exposure) for 16.46% (14.43–18.61) of deaths and 18.01% (15.88–20.21) of DALYs.

**Fig. 6:**
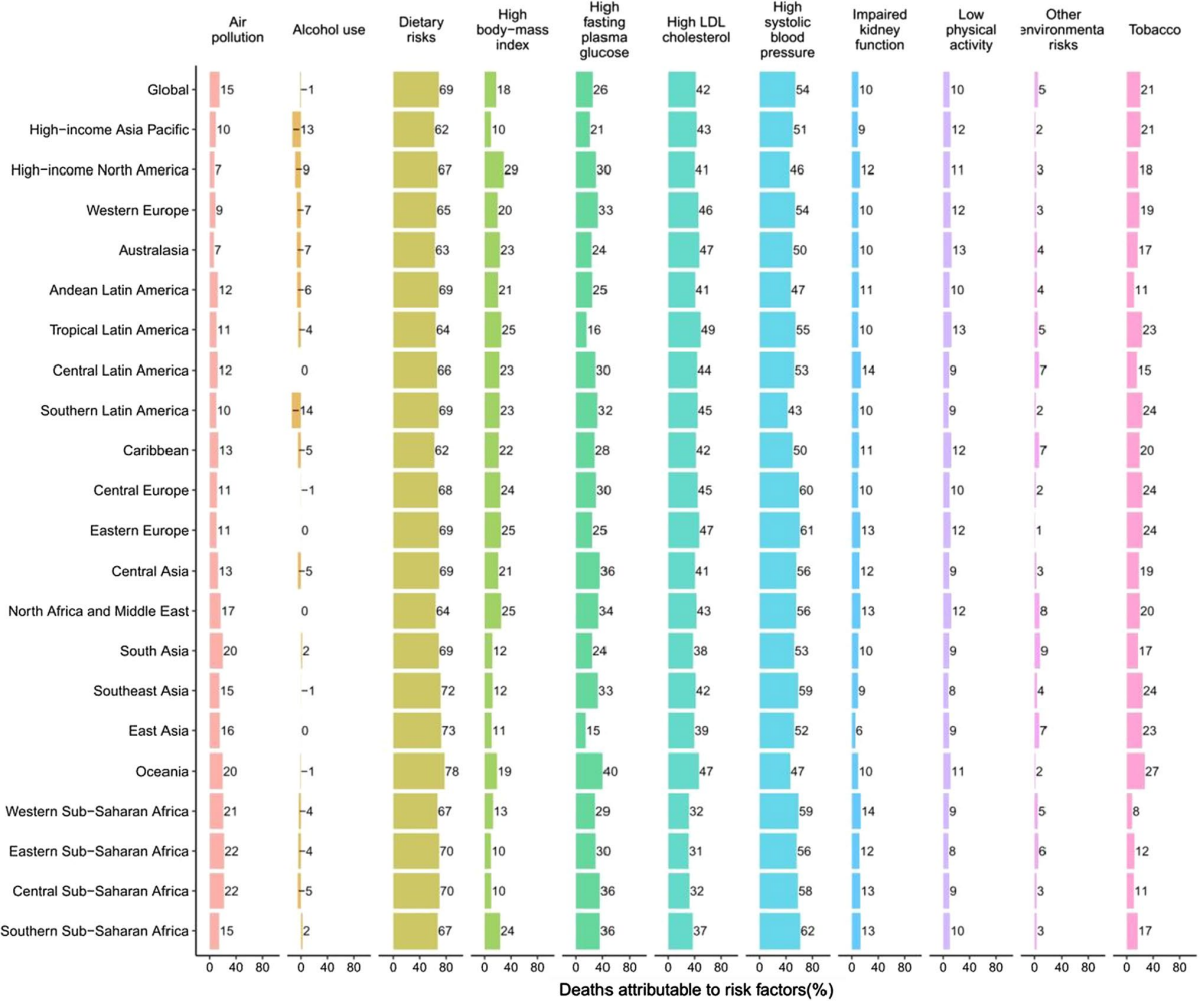
Percentage of age-standardized mortality rate due to ischemic heart disease attributable to risk factors for 21 GBD regions, both sexes, 2017

**Fig. 7:**
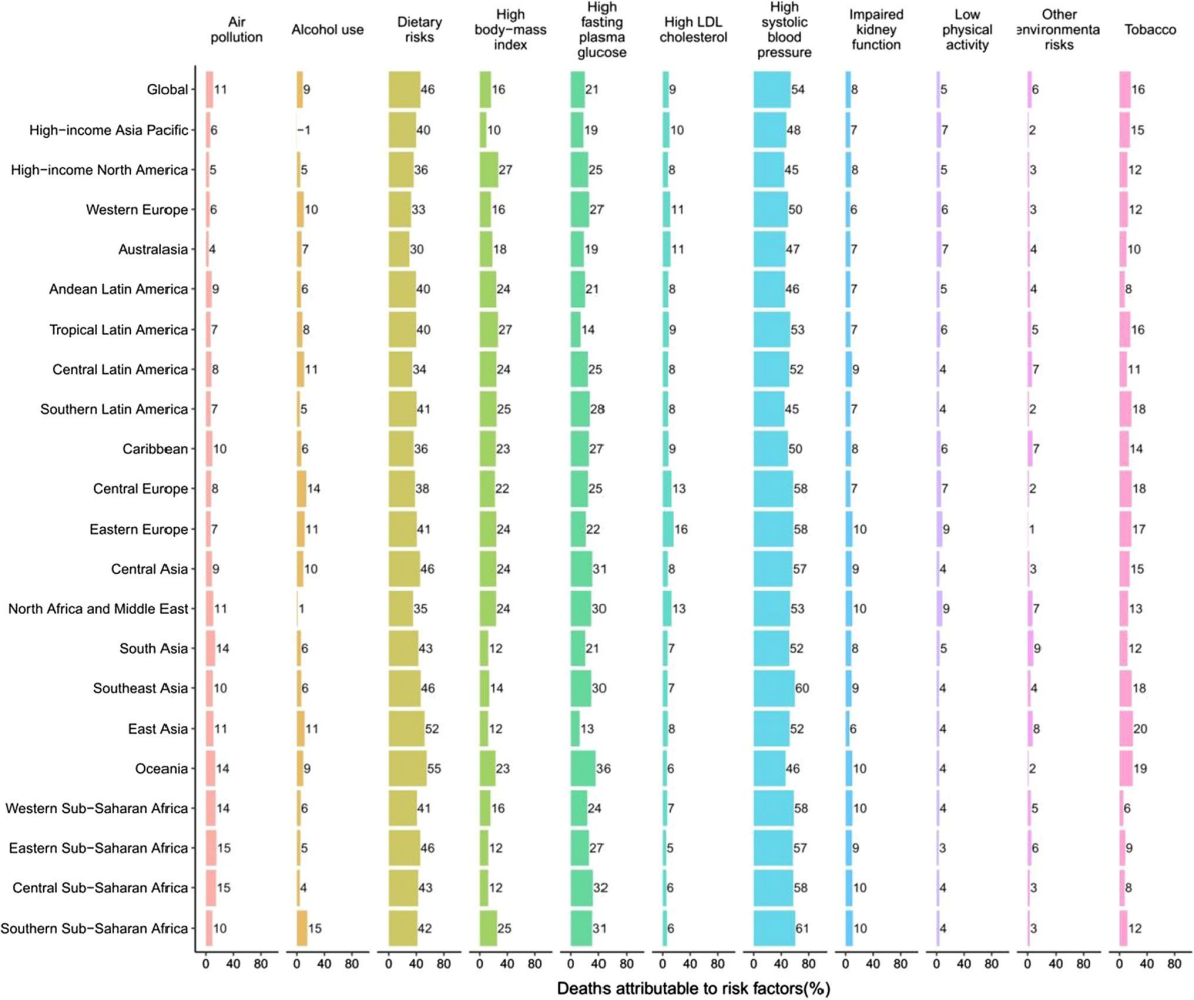
Percentage of age-standardized mortality rate due to stroke attributable to risk factors for 21 GBD regions, both sexes, 2017

Although the proportions of risk factors for deaths and DALYs due to ischemic heart disease differed in the GBD regions, dietary risks, high systolic blood pressure, and high LDL cholesterol had the three highest percentages of attributable age-standardized mortality rates and DALYs for both sexes globally (Fig. 6; supplementary Fig. 10). In 2017, the top three risk factors for global deaths and disability due to ischemic heart disease were dietary risks, high systolic blood pressure, and high LDL cholesterol. For stroke, the proportions of deaths and DALYs attributable to risk factors also differed in the GBD regions, and the three highest percentages of attributable age-standardized mortality rates and DALYs for both sexes globally were high systolic blood pressure, dietary risks, and high fasting plasma glucose (Fig. 7; supplementary Fig. 11). In 2017, the top three risk factors for global deaths and disability due to stroke were high systolic blood pressure, dietary risks, and high fasting plasma glucose.

## Discussion

The global and national burden of CVDs from 1990 to 2017 was reported in detail in this study. While increasing trends for deaths and DALYs due to CVDs were observed, the age-standardized mortality rate and age-standardized rate of DALYs from CVDs declined. It is believed that factors contributing to these declines include improved health care coverage, rapid progress in both prevention and treatment, and improved public health environment [4, 6, 7]. Specifically, ischemic heart disease and stroke, the top two causes of death of CVDs which accounted for about 84% of death of those, showed decreasing trends for age-standardized mortality rate and age-standardized DALY rate from 1990 to 2017. For hypertensive heart disease, fluctuations after a slight decrease were shown in the age-standardized DALY rates and age-standardized mortality rates during the same period.

To examine the factors influencing the changes in mortality and DALYs due to CVDs from 1990 to 2017, the effects of sex, age, social development, and relevant risk factors were analyzed in this study. Differences in the mortality rates due to CVDs between males and females were observed, especially for ischemic heart disease and stroke. This pattern is consistent with other worldwide studies. [6, 13, 14] The reasons for lower mortality of ischemic heart disease and stroke for females are not fully understood so far. The morbidity of ischemic heart disease and stroke was found to be predominant in males, and thus the relatively higher mortality rates observed in males could be related to the higher prevalence of them in males than females. In comparison, the morbidity of hypertensive heart disease in females was slightly higher than that in males.

In 1990 and 2017, mortality from CVDs increased exponentially with age in those aged 15 and older. The prevalence and mortality due to the overall and the three types of CVDs—ischemic heart disease, stroke, and hypertensive heart disease—were largely consistent with age and age-dependent. The increase in life expectancy within the older population itself is raising the number and proportion of the elderly population. Accordingly, the heavy burden of CVDs is very likely to increase with the aging of the worldwide population. Due to the higher prevalence of CVDs, the burden of CVDs will be greater than that of tumor diseases.

In 2017, CVDs were still an important public health issue and a major cause of disease, with a large proportion (about 84.86%) attributable to ischemic heart disease and stroke. Considerable variability in the mortality rates of the overall and the three types of CVDs was found across countries and territories in 2017. Countries and territories with the highest mortality rate had more than a ninefold higher rate of deaths than those with the lowest rate. The countries with the fastest increase in mortality deserve special attention and further support for interventions.

The socio-demographic index level was considered to be a crucial factor influencing the death rate and loss of health, which may explain regional differences [8, 9]. After fluctuation in the expected age-standardized mortality rate of CVDs in most parts of the SDI scale, these rates decrease quickly and constantly for SDI values of 0.7 and higher. Over the same period, most regions have experienced a steady mortality decrease during periods of sustained socio-economic development. But for Eastern Europe, Central Asia, and sub-Saharan Africa, there was an initial spike in mortality rates with increasing SDI, followed by a steady decline. It should be noted that the burden of CVDs is highly associated with a diverse and evolving set of health risks. The dissolution of the Soviet Union and its related effects, including economic crises in the 1990s, were considered to be related to the increase in the burden of CVDs in eastern Europe and central Asia, due to the evidence that the hazardous use of alcohol was the main cause of the situation [6, 15]. For sub-Saharan Africa, the increased burden was accompanied by the “colliding” epidemics of HIV and NCDs [6]. The observed rates could be used to compare with the corresponding expected values to investigate whether the management of CVDs in regions is better or worse than expected. Regional variations in CVDs were likely due to differences in exposure to modifiable risk factors, access to effective health care interventions, and genetic factors.

Although CVDs pose a heavy health burden, our study showed that a considerable part of the deaths and DALYs can be avoided by controlling those metabolic risks and behavioral factors. In our study, metabolic risks accounted for 73.48% (95% UI 68.40–78.12) of deaths and 73.25% (68.98–77.21) of DALYs due to CVDs in both sexes combined globally in 2017, whereas behavioral factors accounted for 63.23% (59.93–66.44) of deaths and 66.71% (63.69–69.28) of attributable DALYs. Considering the interaction among the factors, the best way to avoid CVDs for individuals is to eat healthy, control weight, and reduce smoking. Governments and health institutions should pay more attention to related health education activities and health management of regular monitoring data of hypertension, hyperglycemia, and hyperlipemia for individuals. In addition, since many risk factors for CVDs are treatable (e.g., hypertension, high fasting plasma glucose, and high cholesterol level), appropriate screening and treatment is an important primary prevention strategy.

An unhealthy diet is a significant risk factor for CVDs. Although sodium, sugar, and fat have been the main focus of diet policy debate over the past decades, [16, 17] our study indicated that 69.18% (95% UI 61.66–76.57) of deaths and 73.54% (66.50–80.43) of DALYs from ischemic heart disease were attributable to dietary risks in both sexes combined globally in 2017, whereas there were 46.15% (40.82–51.07) of deaths and 54.79% (48.99–60.41) of DALYs due to those for stroke. Specifically, diet low in nuts and seeds, in whole grains, and in seafood omega-3 fatty acids were the top three dietary risks for ischemic heart disease; diet high in sodium, low in fruits, and in whole grains were for stroke. This finding proposes that, for CVDs, dietary policies focusing on promoting the intake of components of diet for which current intake is less than the optimal level might have a greater effect than policies only targeting sugar and fat [18].

Hypertension is a crucial global health challenge due to its high prevalence and ability to cause stroke [19]. According to data from 30 studies, it is the most common risk factor for stroke [20]. A 19-year cohort study showed that hypertension has a significant effect on the residual lifetime risk of stroke [21]. In addition, hypertension can cause CVDs other than stroke, such as ischemic heart disease. Epidemiological evidence showed that there is a close and consistent link between hypertension and ischemic heart disease [22, 23]. In our study, 53.91% (95% UI 45.59–61.67) of deaths and 56.52% (49.02–63.24) of DALYs from stroke were due to high systolic blood pressure in both sexes combined globally in 2017, whereas there were 54.72% (44.87–64.56) of deaths and 55.65% (48.05–63.10) of DALYs due to it for ischemic heart disease. Previous studies estimated that 26.4% of the world's adult population had hypertension in 2000 [24]. The prevalence and absolute burden of hypertension have been increasing worldwide, especially in low- and middle-income countries [25], so it is anticipated that hypertension will contribute to more deaths and DALYs from stroke and ischemic heart disease in future.

Diabetes has also been associated with an excess risk of ischemic heart disease and stroke in several studies [26–29]. We found that 25.53% (95% UI 14.68–40.86) of deaths and 23.51% (15.23–35.07) of DALYs from ischemic heart disease were due to high fasting plasma glucose in both sexes combined globally in 2017, whereas there were 20.53% (13.39–31.93) of deaths and 20.21% (14.29–28.06) of DALYs due to that for stroke. A collaborative meta-analysis of 102 prospective studies reported that adjusted hazard ratios for ischemic heart disease with diabetes was 2.00, for ischemic stroke was 2.27, and for hemorrhagic stroke was 1.56 [30]. In all countries during 1980–2014, the prevalence of adults diabetes either rose, especially in low and middle SDI areas, or remained unchanged at best [31]. Therefore, the greater contribution of diabetes to ischemic heart disease and stroke in future should not be ignored.

The prevalence of obesity and overweight is increasing at a disturbing rate in a lot of areas around the world [32]. A pooled analysis predicted that global obesity prevalence will be 18% in males and 21% in females by 2025; among them, severe obesity is likely to reach 6% in males and 9% in females [33]. We found that 17.65% (95% UI 10.61–25.92) of deaths and 22.67% (14.48–31.87) of DALYs from ischemic heart disease were due to high BMI in both sexes combined globally in 2017, whereas there were 16.38% (10.01–23.52) of deaths and 24.55% (15.87–33.63) of DALYs due to it for stroke. Although high BMI poses a modest risk for ischemic heart disease and stroke, its consistent rapid increase makes it a more serious risk factor for the future, especially in women, who are more commonly obese than men [31].

High cholesterol is a well-established risk factor for ischemic heart disease. But for stroke, the connection between it and cholesterol is somehow complicated because their relationship changes with the stroke type and cholesterol type. For ischemic stroke, the risk factors are basically the same as those for ischemic heart disease, including high cholesterol. However, for hemorrhagic stroke, which is caused by the rupture of a blood vessel and bleeding into the brain, elevated cholesterol actually reduces the risk of stroke to some extent. This is consistent with our results, which showed that only 8.74% (95% UI 3.33–18.00) of deaths and 9.60% (5.70–16.17) of DALYs from stroke were due to high LDL cholesterol in both sexes combined globally in 2017, whereas there were 41.93% (31.69–52.92) of deaths and 47.73% (39.68–56.13) of DALYs due to that for ischemic heart disease.

### Central Illustration and the Study Limitations

This study provides comprehensive estimates of mortality and DALYs due to CVDs by age, sex, region, and disease from 1990 to 2017. To study the explanatory factors for the contemporary regional variations in mortality and DALYs and the unequal distribution of improvements during the period, the attribution of risk factors, including the SDI and various risk exposures, was investigated.

This study shares the general limitations of all GBD studies [2, 6, 8, 9]. First, although GBD estimates have taken some key steps to improve the reliability and comparability of vital registration data, including redistribution of garbage codes, certain systematic biases may still remain. Second, civil registration and statistics systems are major sources of vital statistics for mortality rates, but population coverage with these systems has been far from desirable, which may affect the quality and representativeness of data. Thirdly, similar to other GBD studies, there is the possibility of ecological fallacy because the interpretation of results from the population level does not necessarily apply to individuals. Therefore, the relevant hypotheses of this study still need to be further confirmed in future individual-based investigations. In addition, there were some other limitations to this study. First, it should be noted that this study was limited to analyses of the overall CVDs and separately of the three main CVDs. The other remaining CVDs were not analyzed separately in this study due to space limitations. Therefore, more efforts are desired for further in-depth study on them. Second, the diagnosis of certain CVDs may be hampered by the incomplete medically certified cause of death system in middle- and low-income countries. The verbal autopsy cause of death data was a useful alternative for the findings presented in GBD studies. Estimates were reported with an uncertainty interval that should always be carefully considered, given that it represents both data sparsity and differences in sample size across data sources and study locations.

### Conclusion and Policy Implications

This study demonstrated that from 1990 to 2017, the number of global deaths and DALYs from CVDs increased, whereas the age-standardized mortality rate and age-standardized DALY rate decreased, with higher mortality and DALYs in males than females. Overall, after fluctuation in the expected age-standardized mortality rate of CVDs in most parts of the lower SDI scale, these rates decrease rapidly for SDI values of 0.7 and higher. Aging and risk exposures, including dietary risks, high systolic blood pressure, high fasting plasma glucose, and high LDL cholesterol, are the main drivers of mortality and health loss. More attention should be paid to them with supportive health policies.

## Perspective

CVDs remain a major global health burden due to increment in death numbers and DALYs, and there is wide regional variation. Aging and the main risk factors are the main drivers of mortality and health loss. More attention should be paid to them with supportive health policies.

## Supplementary Information

Below is the link to the electronic supplementary material.

Supplementary file1 (PDF 2039 kb)

## Data Availability

The datasets generated during and/or analyzed during the current study are available in the Global Health Data Exchange repository, [http://ghdx.healthdata.org/].

## Abbreviations

CVDs: Cardiovascular diseases
NCDs: Non-communicable diseases
DALYs: Disability-adjusted life years
GBD: Global burden of diseases
ICD-10: The international classification of diseases, tenth revision
SDI: Socio-demographic index
UI: Uncertainty intervals
EAPC: The estimated annual percentage change
