# Supplementary material for: Systematic Analysis of the Global, Regional and National Burden of Cardiovascular Diseases from 1990 to 2017
Source: J Epidemiol Glob Health. 2021 Dec 13;12(1):92–103. doi: 10.1007/s44197-021-00024-2 (PMC8907368; doi:10.1007/s44197-021-00024-2)
Supplement: Supplementary file 1 — Supplementary file1 (PDF 2039 kb) [file 44197_2021_24_MOESM1_ESM.pdf]

Systematic Analysis of the Global, Regional and National Burden of Cardiovascular Diseases from 1990 to 2017

Zhenkun Wang[1], PhD, Aihua Du[1]*, PhD, Hong Liu[1], BS, Ziwei Wang[1], MS, Jifa Hu[1,2]*, MS

Affiliations

1. Tongji Hospital, Tongji Medical College, Huazhong University of Science and Technology, Wuhan, 430030, China
2. The Central Hospital of Wuhan, Tongji Medical College, Huazhong University of Science and Technology, Wuhan 430014, China

Correspondence

Aihua Du, Phd
Department of Scientific Research, Tongji Hospital, Tongji Medical College, Huazhong University of Science and Technology, Wuhan, 430030, China.
Email: ahdu@tjh.tjmu.edu.cn
or
Jifa Hu, MS
Department of Scientific Research, Tongji Hospital, Tongji Medical College, Huazhong University of Science and Technology, Wuhan, 430030, China.
Email: jfahu@tjh.tjmu.edu.cn

Supplemental Tables and figures

Supplementary table 1 List of International Classification of Diseases (ICD) codes

Cause	ICD-10
Cardiovascular diseases	B33.2, G45-G46.8, I01-I01.9, I02.0, I05-I09.9, I11-I11.9, I20-I25.9, I28-I28.8, I30-I31.1, I31.8-I37.8, I38-I41.9, I42.1-I42.8, I43-I43.9, I47-I48.9, I51.0-I51.4, I60-I63.9, I65-I66.9, I67.0-I67.3, I67.5-I67.6, I68.0-I68.2, I69.0-I69.3, I70.2-I70.8, I71-I73.9, I77-I83.9, I86-I89.0, I89.9,I98, K75.1
Ischemic heart disease	I20-I25.9
Stroke	G45-G46.8, I60-I63.9, I65-I66.9, I67.0-I67.3, I67.5-I67.6, I68.1-I68.2, I69.0-I69.3
Intracerebral hemorrhage	I61-I62, I62.1-I62.9, I68.1-I68.2, I69.1-I69.2
Ischemic stroke	G45-G46.8, I63-I63.9, I65-I66.9, I67.2-I67.3, I67.5-I67.6, I69.3
Subarachnoid hemorrhage	I60-I60.9, I62.0, I67.0-I67.1, I69.0
Hypertensive heart disease	I11-I11.9

Supplementary table 2 The age-standardized mortality rates of cardiovascular diseases for both sexes combined in 2017

Country/ Territory	Cardiovascular diseases			Ischemic heart disease			Stroke			Hypertensive heart disease		
	Estimate value	Lower 95% UI	Upper 95% UI	Estimate value	Lower 95% UI	Upper 95% UI	Estimate value	Lower 95% UI	Upper 95% UI	Estimate value	Lower 95% UI	Upper 95% UI
Afghanistan	597.03	540.30	650.60	360.01	321.89	405.10	165.80	144.34	188.19	43.17	18.70	58.63
Albania	304.20	262.75	350.42	146.26	124.58	171.26	121.23	103.37	140.02	3.23	2.59	4.40
Algeria	278.36	265.74	291.01	172.38	158.96	189.30	67.76	59.36	76.07	21.53	8.61	31.38
American Samoa	283.75	258.62	308.29	139.09	126.10	152.66	89.60	81.27	98.25	12.40	10.66	14.91
Andorra	109.13	99.25	120.83	53.84	47.69	61.14	27.64	24.19	31.46	2.16	1.65	2.75
Angola	276.04	243.26	313.21	115.05	97.83	135.82	94.08	81.00	108.76	32.96	19.91	46.17
Antigua and Barbuda	191.51	181.07	201.45	75.58	70.56	80.81	59.35	55.25	63.76	22.03	16.67	25.19
Argentina	191.03	174.88	208.98	97.23	88.87	106.42	43.26	39.07	47.70	11.04	9.36	14.60
Armenia	341.01	331.11	351.01	236.55	228.69	248.16	64.51	61.62	67.88	15.08	4.63	17.77
Australia	107.79	99.53	117.49	57.48	52.67	62.69	25.25	23.00	27.69	2.35	1.84	2.97
Austria	145.18	138.20	152.33	82.44	77.38	91.14	22.65	21.04	25.14	10.73	4.36	12.45
Azerbaijan	559.81	529.37	593.26	381.76	358.70	406.24	135.10	124.64	146.31	9.56	7.99	13.87
Bahrain	151.69	139.79	164.92	99.58	91.35	107.97	29.27	26.57	32.32	4.23	3.43	6.98
Bangladesh	298.00	269.61	326.22	117.40	105.75	129.79	153.00	138.85	168.15	10.13	7.05	15.30
Barbados	170.05	158.63	180.78	62.94	57.91	68.13	60.01	55.30	64.48	11.93	9.77	15.14
Belarus	443.13	422.29	464.51	318.00	303.19	334.04	95.66	89.93	101.48	6.19	3.92	7.63
Belgium	114.90	108.87	121.22	55.89	52.25	60.17	29.08	27.01	31.54	2.25	1.44	2.77
Belize	176.96	170.55	183.78	79.31	74.96	83.69	50.35	47.38	53.39	17.53	13.65	22.32
Benin	235.85	203.61	272.38	98.67	84.29	114.85	93.77	78.60	110.12	11.76	6.41	17.69

Bermuda	139.55	131.67	148.09	74.12	69.38	79.28	29.90	27.69	32.39	6.43	4.23	7.49
Bhutan	217.07	182.48	254.97	120.38	98.49	143.72	63.39	50.02	78.46	9.77	7.13	12.90
Bolivia	204.30	173.23	236.76	108.75	91.97	128.68	62.14	51.00	73.88	10.94	7.94	14.08
Bosnia and Herzegovina	329.63	313.23	345.95	164.97	153.50	175.39	123.52	114.02	132.08	4.73	3.88	8.40
Botswana	237.37	219.06	263.97	103.36	93.22	116.53	85.43	76.17	96.84	22.05	16.27	28.09
Brazil	177.96	175.92	180.01	80.02	77.92	81.67	56.58	55.21	57.79	10.65	8.82	14.19
Brunei	201.28	186.76	216.07	105.19	96.88	113.93	57.24	52.48	62.22	7.74	6.81	8.98
Bulgaria	424.69	406.94	442.63	220.41	210.09	231.83	135.87	128.83	143.46	36.05	23.05	40.27
Burkina Faso	269.05	242.39	295.84	135.87	121.88	151.56	79.03	70.16	88.66	17.01	8.99	22.77
Burundi	293.07	255.97	332.58	125.82	106.05	147.35	104.30	83.61	126.41	38.25	15.82	73.16
Cambodia	270.89	249.80	300.03	81.39	72.82	91.94	153.99	139.71	171.81	15.96	9.74	20.57
Cameroon	244.66	208.23	283.52	101.03	81.59	121.12	94.66	78.48	114.05	12.94	6.86	18.82
Canada	105.60	100.78	110.62	63.34	60.19	66.61	23.03	21.65	24.45	1.58	1.02	1.79
Cape Verde	182.22	170.20	193.35	101.98	93.90	110.21	47.69	41.71	53.81	6.31	5.45	8.02
Central African Republic	435.73	363.78	497.71	188.86	156.64	219.93	156.48	127.71	186.50	46.62	24.14	67.78
Chad	281.00	248.26	315.89	124.76	104.67	147.03	109.46	92.71	128.49	12.75	6.90	18.38
Chile	127.99	117.03	139.49	52.47	47.72	57.93	43.82	39.83	48.13	11.00	6.84	12.69
China	261.90	253.38	270.03	107.22	103.90	110.77	122.41	118.65	126.65	18.63	11.70	20.70
Colombia	124.24	113.67	135.38	71.20	64.75	77.72	28.71	25.67	31.87	8.88	7.45	15.02
Comoros	261.52	228.10	298.95	130.54	109.99	153.22	86.57	73.38	103.41	23.00	12.70	36.94
Congo	344.09	298.25	388.10	159.33	135.35	183.96	109.92	92.66	129.91	36.15	19.86	50.78
Costa Rica	137.97	130.15	146.51	77.86	72.42	83.75	30.36	27.92	32.95	11.52	8.83	13.36

Cote d'Ivoire	303.74	268.17	341.28	148.86	129.86	168.79	109.24	94.66	124.92	11.95	6.47	17.96
Croatia	253.78	241.30	266.80	142.41	134.82	152.06	72.23	67.74	77.57	14.53	8.36	16.31
Cuba	190.97	174.11	208.84	107.16	97.72	117.68	50.44	45.52	56.06	9.98	5.33	11.61
Cyprus	141.17	130.91	154.29	79.02	72.13	89.33	30.83	28.03	35.21	5.73	2.70	6.78
Czech Republic	227.48	216.40	239.05	147.12	139.97	155.20	48.33	45.35	51.26	5.76	4.45	7.10
Democratic Republic of the Congo	318.95	279.93	359.60	143.08	120.70	167.42	107.58	89.95	126.24	37.29	20.71	61.17
Denmark	114.77	109.03	121.31	54.52	51.30	58.40	32.25	30.15	34.76	1.91	1.32	2.17
Djibouti	258.04	203.03	322.02	127.98	98.41	161.71	84.22	65.09	107.35	22.70	12.06	33.64
Dominica	227.38	214.91	240.49	77.95	72.75	83.81	59.66	55.34	64.68	33.99	27.95	42.29
Dominican Republic	266.65	236.69	296.55	151.32	133.74	169.62	78.47	69.09	88.47	16.07	13.45	18.69
Ecuador	140.45	130.01	151.50	66.42	60.67	72.30	39.62	36.05	43.23	18.14	13.51	21.85
Egypt	525.43	480.49	576.25	350.68	313.60	388.52	120.83	107.40	135.90	32.52	14.12	42.43
El Salvador	167.29	145.71	192.16	109.59	95.19	125.63	34.24	29.58	39.62	8.86	7.15	12.84
Equatorial Guinea	202.81	149.34	264.27	82.22	60.54	108.34	61.46	44.25	81.88	26.38	14.73	38.62
Eritrea	311.11	264.76	356.14	121.46	99.77	143.65	117.34	95.52	139.37	40.19	22.04	64.74
Estonia	255.57	229.61	281.06	141.09	121.76	184.28	37.09	31.75	48.81	51.93	9.76	64.95
Ethiopia	182.63	165.49	203.91	82.60	69.39	96.52	62.39	52.44	73.51	19.86	10.72	31.72
Federated States of Micronesia	454.34	405.26	508.81	223.37	198.99	251.18	156.33	136.41	177.90	23.88	17.04	29.99
Fiji	412.82	369.71	456.53	226.65	202.32	252.61	92.13	81.66	103.19	30.53	26.17	36.17
Finland	153.51	146.46	162.08	88.60	83.33	96.69	32.53	30.42	35.27	7.15	2.83	8.35
France	86.06	81.81	90.86	38.27	35.97	41.35	21.34	19.93	23.13	2.80	1.54	3.20
Gabon	259.97	230.71	290.54	121.25	105.50	137.57	77.25	67.33	89.58	27.59	14.88	36.99

Georgia	496.22	483.49	509.20	249.46	238.40	267.05	167.07	159.16	180.46	46.22	17.99	53.45
Germany	156.14	143.03	169.06	84.76	77.40	92.99	28.62	25.95	31.59	10.02	5.79	11.55
Ghana	298.25	268.85	331.05	131.60	117.13	147.12	122.89	107.98	138.81	10.27	5.44	15.98
Greece	175.69	166.87	184.48	92.48	87.56	97.31	54.88	51.52	58.47	6.08	3.11	6.98
Greenland	199.94	188.02	213.76	90.50	83.97	98.78	69.65	63.84	76.25	4.79	3.94	5.72
Grenada	243.96	231.75	257.07	103.93	97.88	110.68	78.11	73.24	83.62	19.16	13.76	22.46
Guam	310.50	291.42	330.91	202.44	186.96	217.95	61.90	56.81	67.01	18.31	15.74	26.63
Guatemala	155.90	144.47	168.60	92.46	84.54	100.42	43.59	39.87	47.71	8.24	6.88	13.71
Guinea	336.72	302.07	371.70	157.44	139.93	177.29	130.91	114.24	149.23	13.38	6.95	20.24
Guinea-Bissau	382.47	337.78	431.49	173.82	151.44	197.96	148.10	128.91	169.73	17.28	8.91	25.78
Guyana	373.16	339.72	408.08	166.62	151.39	183.11	127.47	115.29	141.07	39.79	33.75	52.35
Haiti	430.55	371.94	494.70	185.39	154.73	217.73	153.45	128.53	180.42	37.39	24.10	47.96
Honduras	240.21	200.50	279.85	152.56	124.38	178.12	56.49	45.83	67.18	15.05	11.25	22.89
Hungary	278.30	266.93	290.61	165.45	157.83	174.30	57.12	53.86	60.63	22.33	13.44	24.73
Iceland	117.99	113.60	122.59	71.10	68.31	74.17	23.49	22.15	24.92	2.98	1.69	3.41
India	282.28	264.98	293.33	164.68	155.07	171.22	77.42	72.74	81.10	11.86	8.84	14.69
Indonesia	342.86	324.36	364.73	131.51	123.82	140.29	178.32	167.60	189.75	14.70	12.03	17.51
Iran	270.31	266.67	277.82	159.53	155.90	169.28	61.28	59.18	65.12	29.53	23.71	31.28
Iraq	218.61	206.66	231.50	129.73	122.11	138.39	67.15	63.09	71.67	7.48	6.59	9.97
Ireland	126.46	119.01	134.12	70.90	66.52	76.17	27.91	25.96	30.01	2.07	1.48	2.48
Israel	93.32	88.10	98.95	47.65	44.83	50.73	23.92	22.32	25.72	1.74	1.42	3.45
Italy	113.15	107.71	119.08	51.39	47.91	56.56	30.42	28.32	33.75	10.61	4.12	12.37
Jamaica	206.54	184.31	231.51	70.74	62.01	80.18	92.65	81.09	105.69	18.68	14.92	27.10
Japan	79.37	77.13	81.43	32.97	31.82	34.07	30.01	29.05	30.96	2.09	1.78	4.17

Jordan	208.26	190.04	229.62	104.12	93.41	117.62	58.66	52.63	66.81	31.54	21.79	36.52
Kazakhstan	466.79	449.03	484.10	275.19	263.95	287.19	135.34	127.77	142.97	10.67	9.00	19.49
Kenya	218.64	199.34	242.78	88.98	77.02	105.79	84.53	72.00	98.63	22.75	12.84	32.19
Kiribati	434.66	385.78	482.72	182.90	160.57	205.55	182.00	160.62	204.00	22.28	17.56	26.78
Kuwait	132.24	124.54	140.07	80.96	75.33	87.12	30.00	27.81	32.54	11.67	9.99	16.97
Kyrgyzstan	436.36	425.94	447.95	293.14	284.86	302.59	115.06	110.30	119.93	10.34	8.58	13.34
Laos	368.11	328.18	416.85	156.03	136.59	178.43	169.23	148.32	191.07	17.57	12.01	22.11
Latvia	350.06	319.57	382.08	198.03	180.28	220.29	100.12	89.87	111.92	14.02	3.36	17.44
Lebanon	266.59	252.04	280.87	192.17	180.14	205.98	41.40	36.82	47.38	18.30	7.57	26.88
Lesotho	405.13	332.82	476.05	146.83	117.92	177.56	172.94	139.40	209.71	43.86	29.71	57.93
Liberia	272.51	237.71	311.24	138.71	119.60	159.23	94.56	81.42	108.92	10.63	6.31	15.28
Libya	341.86	307.19	380.72	234.17	205.61	263.40	71.12	55.83	85.76	18.89	7.84	27.50
Lithuania	342.99	326.08	360.31	233.16	221.42	245.76	74.66	70.19	79.37	7.33	3.65	8.35
Luxembourg	128.28	116.92	139.94	60.57	54.13	67.09	30.24	26.87	34.03	3.79	2.28	4.56
Macedonia	322.69	307.71	337.96	125.91	117.60	134.36	149.79	140.28	159.83	20.20	13.78	23.85
Madagascar	405.99	352.25	465.30	136.27	116.28	159.45	181.95	153.44	213.64	46.50	24.16	75.85
Malawi	227.35	202.48	252.51	103.72	89.64	119.03	79.40	68.41	91.37	24.80	12.76	44.27
Malaysia	260.94	241.54	283.57	149.77	137.60	163.79	83.74	76.41	91.78	2.36	1.98	3.60
Maldives	164.91	154.31	175.56	102.70	95.53	110.18	40.71	37.07	44.26	6.70	5.69	7.87
Mali	268.02	237.24	300.04	114.51	96.99	133.40	107.12	90.84	127.43	11.98	6.95	17.71
Malta	168.71	161.72	175.78	104.33	99.53	109.22	35.83	33.74	38.01	4.63	3.12	5.23
Marshall Islands	557.79	505.45	618.43	261.90	232.60	293.67	201.20	177.56	229.11	30.89	20.04	38.78
Mauritania	232.35	203.05	262.50	116.21	99.44	133.87	79.20	67.51	91.58	9.07	5.14	13.51
Mauritius	224.64	212.64	236.79	117.14	109.39	124.61	68.08	63.83	73.16	23.60	16.04	26.43

Mexico	152.78	149.78	156.18	95.85	92.31	98.80	34.47	33.16	35.44	8.80	7.24	11.59
Moldova	408.50	398.12	419.03	267.90	258.20	286.60	101.33	96.52	108.61	23.67	4.98	28.26
Mongolia	460.04	427.58	497.53	248.23	228.57	269.16	177.86	163.29	194.33	9.01	7.62	10.50
Montenegro	387.30	364.81	411.89	149.60	139.13	161.63	185.15	172.47	198.69	2.24	1.64	2.63
Morocco	419.15	365.47	476.45	276.00	237.59	318.44	99.80	80.93	119.26	24.80	10.18	33.16
Mozambique	329.94	289.19	371.13	111.10	94.77	128.24	153.35	130.58	179.29	37.29	20.73	60.59
Myanmar	202.10	184.32	220.69	84.14	75.12	94.39	92.11	82.98	100.37	10.18	7.60	13.01
Namibia	243.81	217.24	277.16	101.43	89.27	116.11	91.66	79.23	105.82	22.93	18.09	28.92
Nepal	260.80	227.61	292.28	157.30	135.72	177.94	74.12	63.08	85.35	8.76	6.84	11.10
Netherlands	109.36	103.94	115.34	49.51	46.66	52.75	29.33	27.53	31.70	2.27	1.34	2.61
New Zealand	128.80	122.74	135.76	70.57	67.00	74.76	28.41	26.66	30.36	2.52	2.13	4.32
Nicaragua	137.02	125.15	150.26	81.09	73.79	88.92	33.92	30.43	37.78	11.38	9.81	13.13
Niger	238.34	207.18	271.69	98.18	76.28	120.00	98.85	82.28	116.60	11.83	6.21	19.42
Nigeria	181.01	138.95	236.19	79.15	60.17	104.75	68.21	51.63	90.13	8.20	4.78	12.83
North Korea	321.68	280.52	361.45	111.15	91.15	129.59	171.72	148.94	195.25	19.32	13.47	28.32
Northern Mariana Islands	194.99	179.26	213.38	94.36	86.47	103.69	68.59	62.12	76.13	5.56	4.86	6.57
Norway	114.32	111.01	118.73	58.33	56.17	62.09	28.09	26.95	29.92	1.61	1.10	1.78
Oman	266.34	233.26	299.67	183.45	159.15	207.80	57.27	49.53	65.11	3.48	2.62	6.53
Pakistan	423.03	364.84	483.77	235.54	202.45	275.32	133.72	112.66	155.68	18.16	13.89	24.03
Palestine	265.91	250.80	283.29	152.98	142.68	164.35	81.50	75.54	87.45	13.04	11.28	17.30
Panama	128.35	121.63	135.35	61.74	57.76	65.89	42.87	40.16	45.72	6.53	5.72	9.14
Papua New Guinea	561.49	501.23	627.54	267.48	232.96	307.65	198.15	168.07	228.41	27.74	16.60	35.05
Paraguay	199.13	174.52	229.00	99.67	86.82	114.56	66.97	58.30	77.23	13.82	7.56	17.03

Peru	85.75	75.66	96.05	46.84	41.25	52.80	24.62	21.55	27.83	3.25	2.57	3.95
Philippines	370.44	332.91	409.20	183.90	164.29	204.86	118.97	105.63	133.04	42.57	25.46	49.47
Poland	227.33	218.10	237.50	130.81	125.02	136.94	53.16	50.25	56.19	5.64	4.88	9.54
Portugal	127.84	121.37	134.17	48.32	45.19	51.50	53.85	50.66	57.28	4.56	2.17	5.31
Puerto Rico	108.09	102.44	114.06	57.71	54.53	61.45	23.39	21.82	25.14	10.42	7.42	11.93
Qatar	176.69	154.10	202.08	122.88	106.53	141.01	35.29	30.40	40.47	2.49	1.95	4.40
Romania	370.95	356.21	384.97	174.14	166.20	184.03	125.25	119.31	132.63	30.12	18.44	33.31
Russian Federation	431.30	427.42	436.80	240.95	237.59	248.09	135.32	133.04	139.30	7.47	4.49	8.05
Rwanda	191.38	168.84	215.27	61.18	42.74	81.10	76.04	58.21	93.68	31.75	14.73	53.92
Saint Lucia	204.62	193.65	215.25	66.46	61.40	71.39	74.60	69.19	79.70	18.91	14.98	24.23
Saint Vincent and the Grenadines	252.68	240.81	265.44	113.19	106.62	120.03	76.08	71.42	81.06	29.78	23.03	33.62
Samoa	348.98	311.13	380.77	173.69	152.77	193.35	116.56	101.59	131.32	19.08	13.85	24.17
Sao Tome and Principe	270.11	238.21	303.07	121.64	104.61	140.43	102.74	88.20	122.55	4.71	3.01	6.41
Saudi Arabia	259.54	241.07	284.02	167.71	154.91	184.39	68.88	62.23	76.87	2.42	1.87	4.07
Senegal	241.22	215.40	271.06	119.89	105.77	135.74	87.13	76.67	99.13	9.20	5.10	13.81
Serbia	439.42	419.06	462.16	208.23	196.86	222.47	167.93	157.95	179.79	18.05	13.28	20.60
Seychelles	242.65	231.63	253.66	95.60	89.14	102.21	56.13	51.57	60.25	59.85	49.33	72.23
Sierra Leone	325.72	287.69	368.71	161.89	142.02	184.55	116.22	101.42	132.89	12.98	7.12	17.50
Singapore	92.24	87.47	97.58	51.64	48.32	55.30	22.03	20.70	23.70	9.02	5.58	10.25
Slovakia	287.96	274.10	303.90	193.48	183.17	205.87	58.63	54.48	63.25	10.90	6.77	12.61
Slovenia	153.49	144.78	163.62	69.58	64.45	78.89	38.90	35.92	44.01	12.07	5.38	14.19
Solomon Islands	459.78	418.03	503.16	236.20	212.45	260.82	164.14	148.23	181.37	19.42	12.25	24.26

Somalia	365.77	294.10	444.12	162.56	126.10	204.02	136.15	104.47	168.43	38.33	21.04	70.42
South Africa	200.38	194.22	208.59	83.47	79.72	87.90	65.76	62.43	69.71	25.58	22.81	28.50
South Korea	86.00	80.29	91.81	32.03	29.65	34.51	38.87	35.90	41.86	5.70	4.74	8.49
South Sudan	280.78	226.29	347.21	121.03	93.87	153.65	106.24	83.91	132.64	28.13	15.32	48.64
Spain	99.40	94.47	104.46	45.43	42.69	48.76	25.25	23.57	27.35	4.26	1.83	4.99
Sri Lanka	197.09	171.63	220.22	107.93	93.84	122.27	58.48	50.36	66.04	8.41	5.10	10.62
Sudan	431.39	365.33	496.58	263.13	215.89	312.72	114.86	90.50	141.91	30.28	12.34	43.68
Suriname	258.31	236.05	279.72	107.79	97.33	117.33	102.12	92.43	111.37	20.29	17.13	27.34
Swaziland	333.44	278.27	397.54	140.95	115.94	169.96	126.30	104.12	153.29	31.76	22.02	43.62
Sweden	133.98	127.84	140.38	72.41	68.24	79.51	29.53	27.51	32.76	4.57	1.17	5.43
Switzerland	99.74	94.35	105.22	53.39	49.37	60.13	19.51	17.80	22.34	6.71	1.88	8.04
Syria	376.26	333.88	425.39	284.49	251.58	323.02	68.10	59.85	76.91	4.09	3.38	5.87
Taiwan (Province of China)	103.96	99.38	109.20	45.60	43.23	48.21	35.56	33.61	37.54	8.71	7.47	11.26
Tajikistan	427.70	399.22	459.74	268.09	249.10	289.19	111.94	102.91	122.14	32.53	25.07	36.48
Tanzania	217.29	193.37	243.77	102.45	86.65	120.68	65.65	54.92	77.98	26.07	13.84	38.41
Thailand	109.86	100.46	121.55	47.68	43.34	53.25	45.90	41.50	51.49	2.06	1.79	2.35
The Bahamas	235.95	219.99	252.61	90.04	82.89	97.80	60.29	54.74	66.06	47.98	37.44	55.12
The Gambia	331.43	294.84	371.19	170.31	149.73	192.16	114.93	99.11	132.16	12.72	7.76	17.27
Timor-Leste	335.35	293.02	375.86	137.61	108.02	163.32	161.46	136.41	183.82	14.65	10.55	17.90
Togo	280.03	245.67	320.87	135.30	116.81	157.65	102.76	88.48	121.40	11.39	6.62	16.62
Tonga	227.49	203.86	254.29	122.88	110.35	138.07	72.76	63.42	82.47	7.58	6.30	9.11
Trinidad and Tobago	228.47	195.72	266.55	122.05	103.40	142.93	64.77	54.97	75.88	16.50	13.00	27.50
Tunisia	318.99	270.61	372.19	205.05	172.16	240.05	75.06	63.14	89.22	22.31	8.92	31.10

Turkey	171.29	158.75	183.77	98.28	90.13	106.55	44.75	41.22	48.47	13.30	8.84	15.18
Turkmenistan	536.78	508.60	568.63	339.30	319.85	359.68	148.85	139.20	158.98	17.03	13.55	23.90
Uganda	213.33	190.29	238.28	86.58	70.02	103.64	77.83	64.76	91.33	26.73	13.35	40.39
Ukraine	539.85	519.52	562.36	386.14	371.42	402.14	109.90	104.88	115.60	1.69	1.42	2.29
United Arab Emirates	317.84	269.67	370.94	179.50	149.17	212.79	93.25	78.36	110.42	27.39	12.36	38.51
United Kingdom	122.14	120.76	123.61	61.30	60.30	62.53	31.29	30.65	31.98	2.83	2.16	3.40
United States	151.09	148.08	153.90	88.62	86.23	91.07	28.60	27.62	29.53	7.57	4.67	8.15
Uruguay	160.71	147.33	174.83	68.17	62.09	74.53	53.58	48.61	58.77	7.87	5.51	9.29
Uzbekistan	724.42	663.68	785.34	534.24	488.06	579.99	149.14	135.04	162.80	19.83	14.38	24.14
Vanuatu	546.30	449.18	646.94	283.33	220.54	344.27	176.76	141.83	218.61	25.54	17.64	33.95
Venezuela	204.85	182.07	231.13	120.98	105.76	137.87	50.64	44.21	57.75	18.52	15.40	25.93
Vietnam	245.46	227.11	269.41	80.16	73.14	88.80	135.95	124.48	150.01	11.51	8.86	14.14
Virgin Islands, U.S.	273.67	239.85	300.59	154.63	133.36	171.10	50.93	43.90	56.81	24.89	19.91	30.29
Yemen	495.00	402.25	603.12	300.41	243.89	367.73	135.10	107.24	166.66	34.58	14.08	46.93
Zambia	234.50	209.50	258.67	107.73	95.93	120.31	81.17	71.26	91.94	23.39	12.73	33.06
Zimbabwe	307.85	272.20	348.74	164.80	143.07	186.88	82.84	71.84	94.65	13.59	8.67	26.60

UI, uncertainty interval.

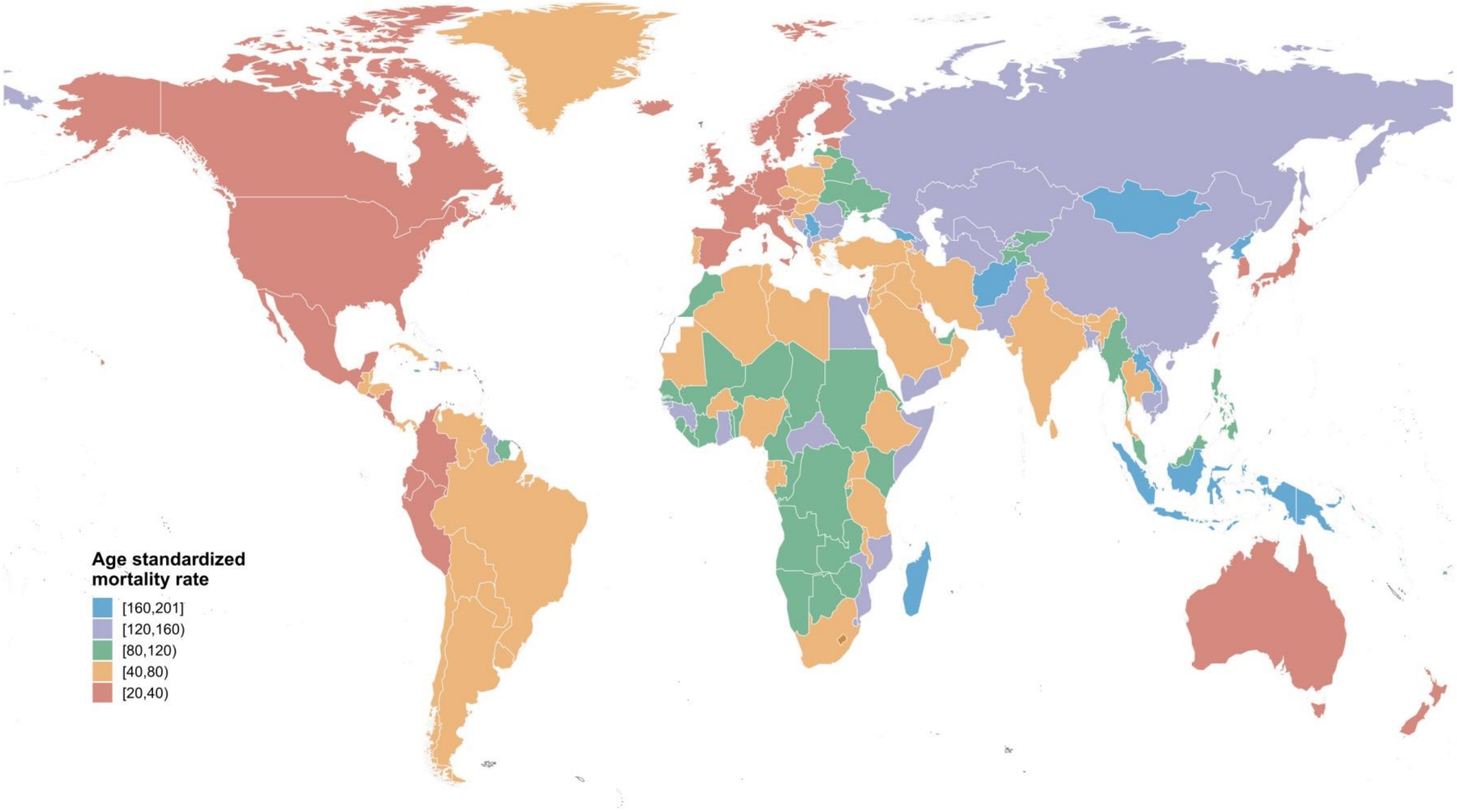

Supplementary fig 1 Global age-standardized mortality rate per 100,000 people of stroke for both sexes combined in 195 countries and territories in 2017

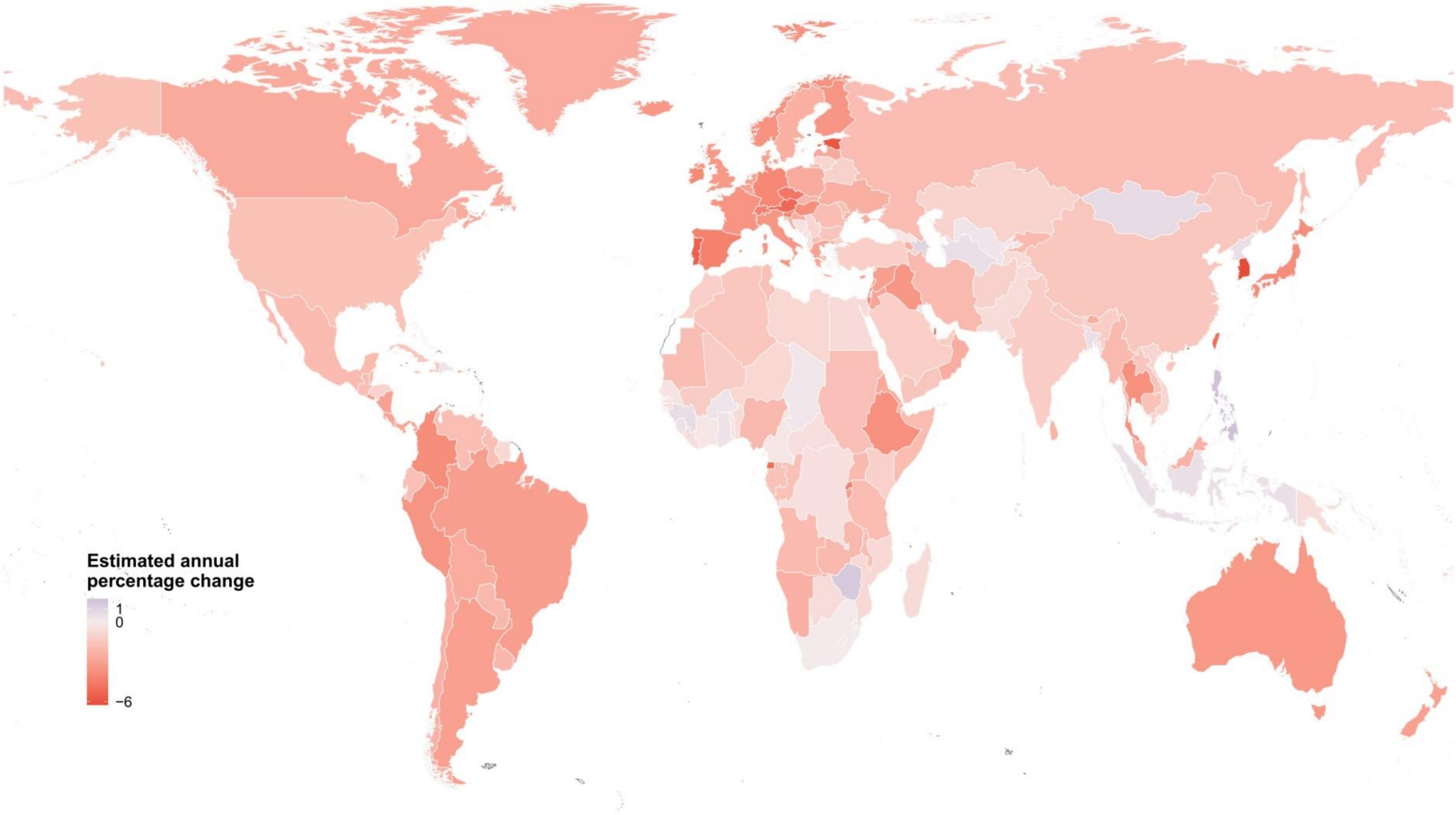

Supplementary fig 2 Estimated annual percentage change in age-standardized mortality rate per 100,000 people of stroke for both sexes combined in 195 countries and territories from 1990 to 2017

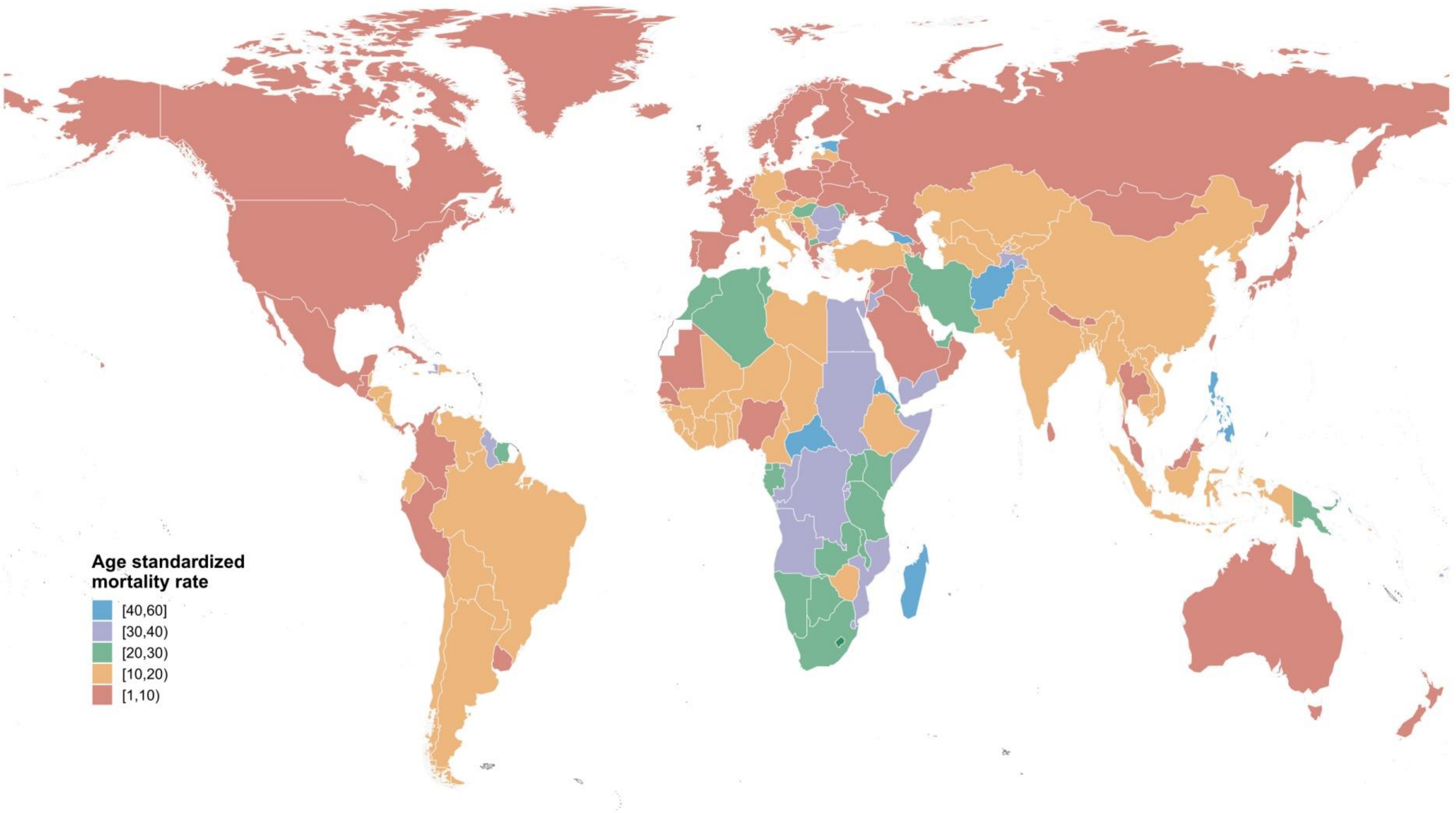

Supplementary fig 3 Global age-standardized mortality rate per 100,000 people of hypertensive heart disease for both sexes combined in 195 countries and territories in 2017

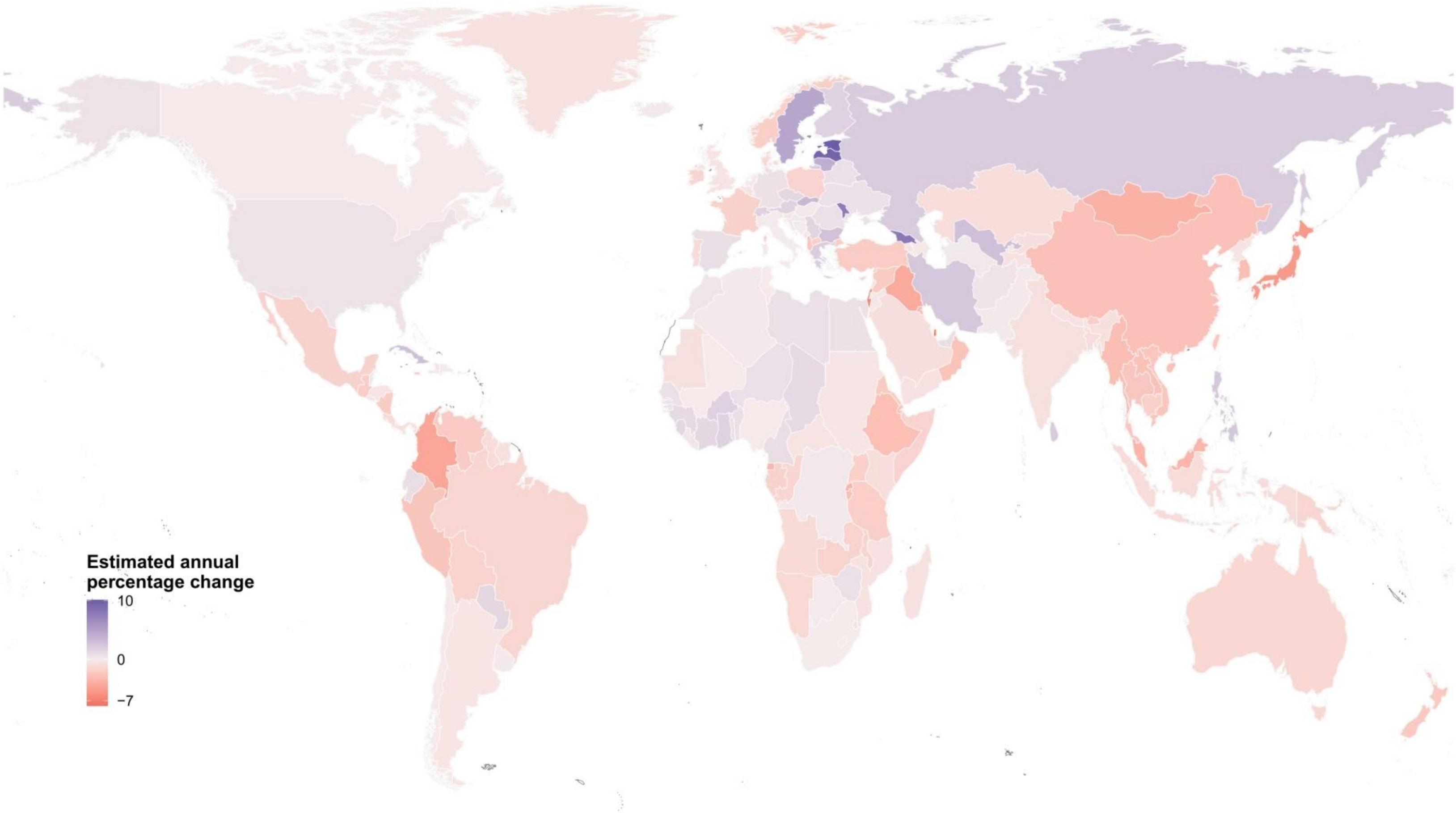

Supplementary fig 4 Estimated annual percentage change in age-standardized mortality rate per 100,000 people of hypertensive heart disease for both sexes combined in 195 countries and territories from 1990 to 2017

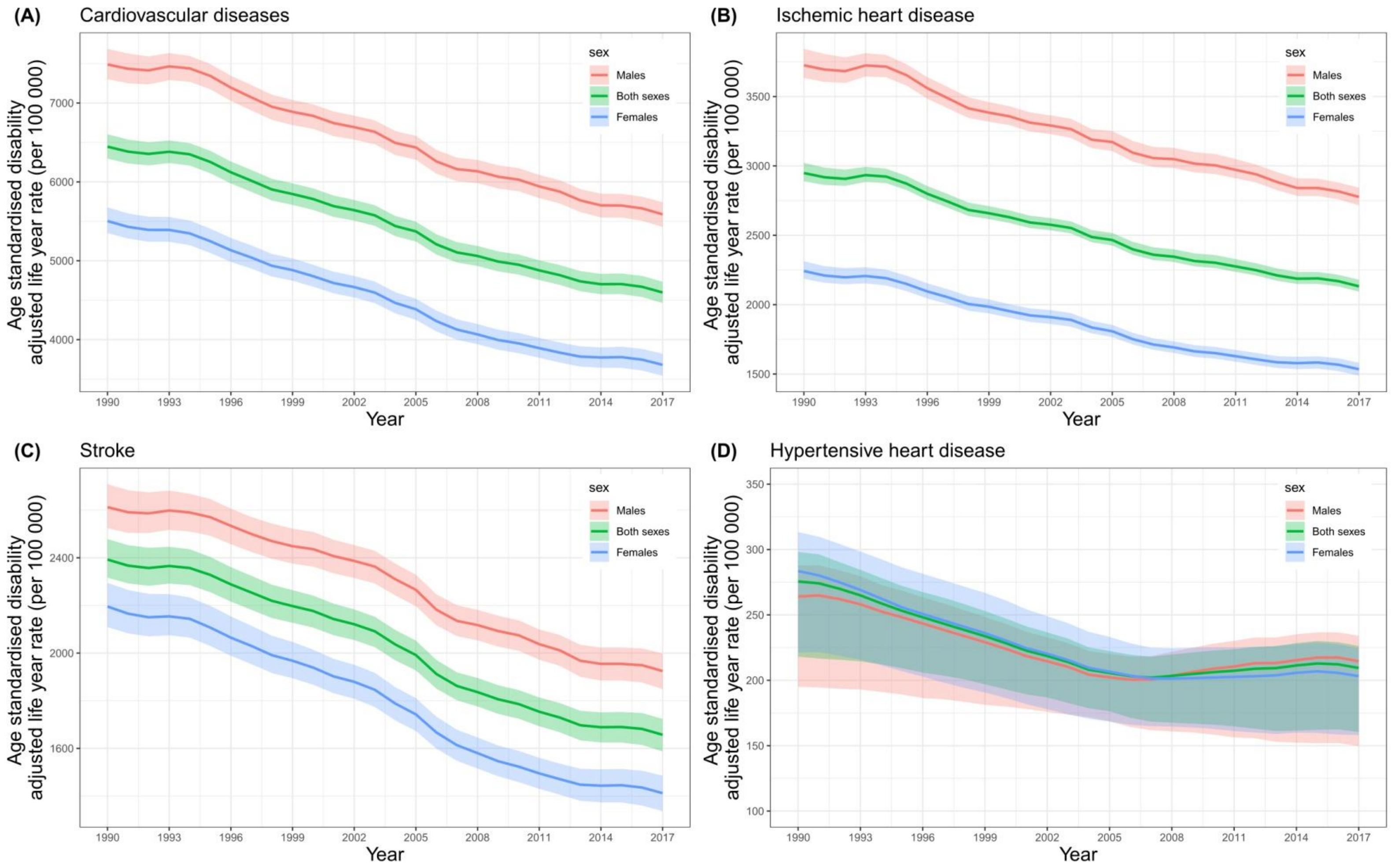

Supplementary fig 5 Age-standardized DALY rate per 100,000 people of cardiovascular diseases; ischemic heart disease; stroke; and hypertensive heart disease in males, females, and both sexes from 1990 to 2017. Shading indicates 95% uncertainty intervals

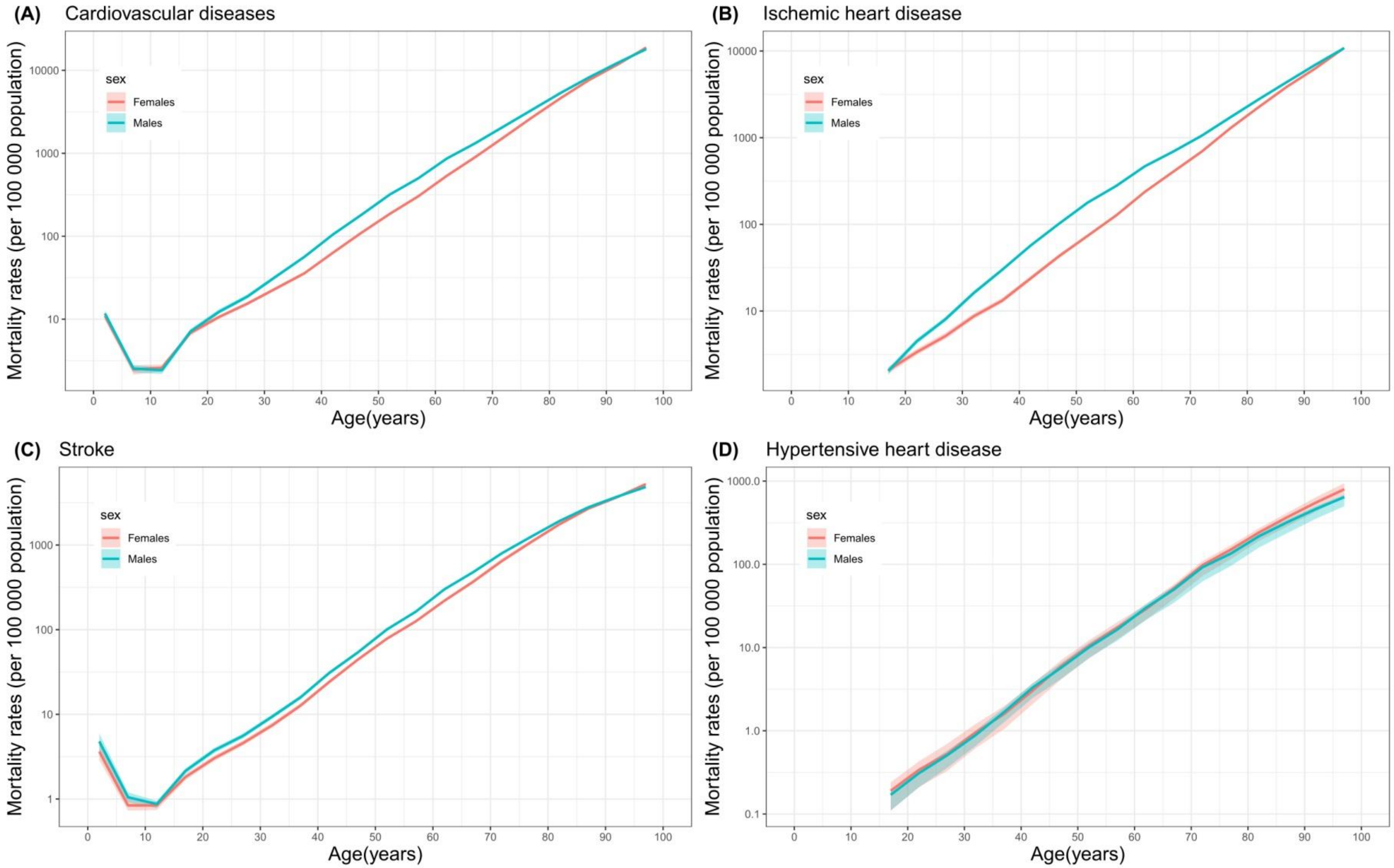

Supplementary fig 6 Global mortality rates per 100,000 people of cardiovascular diseases; ischemic heart disease; stroke; and hypertensive heart disease by age in males and females in 1990. The y axis is represented on a logarithmic scale. Shading indicates 95% uncertainty intervals

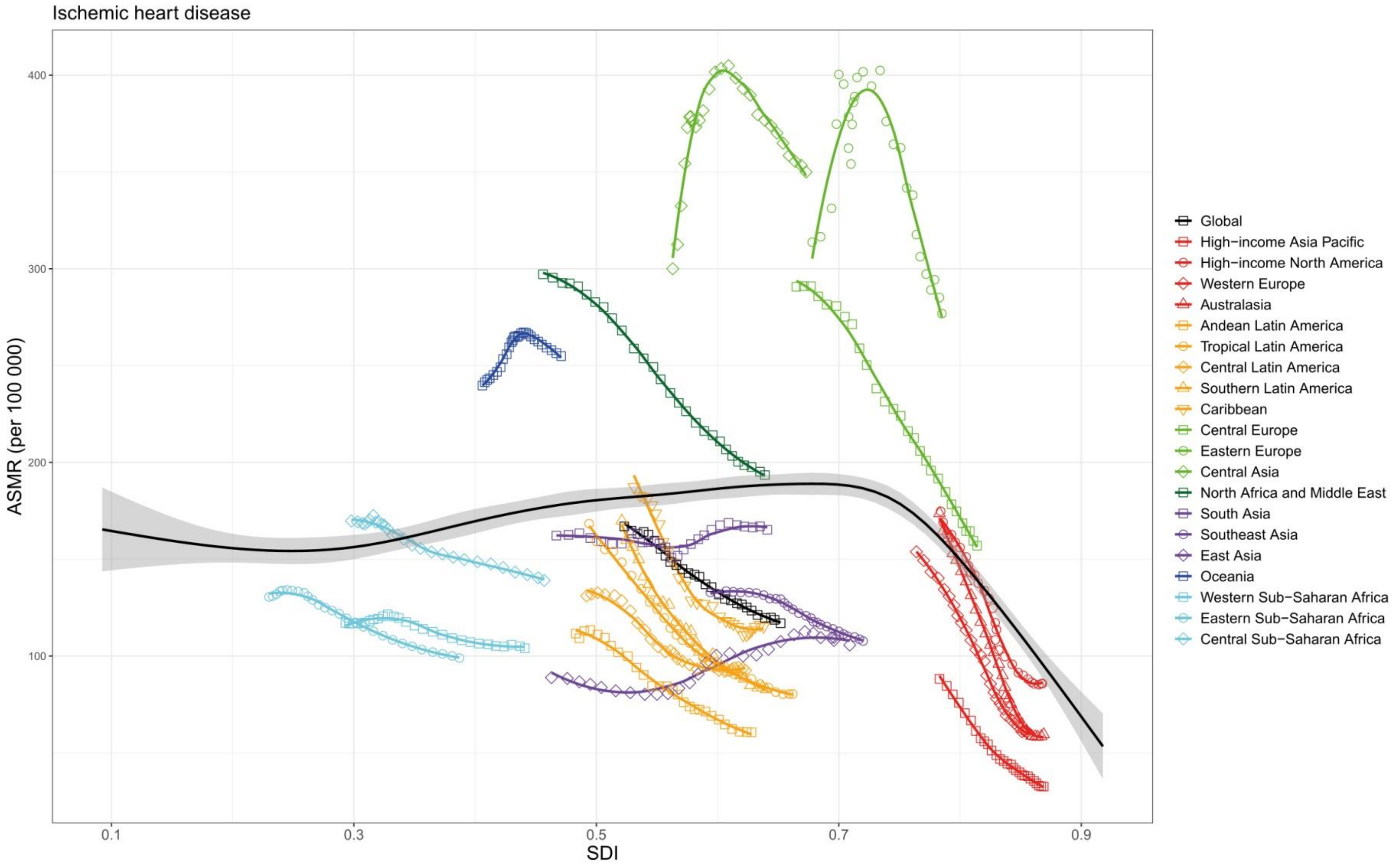

Supplementary fig 7 Age-standardized mortality rate of ischemic heart disease for 21 GBD regions, 1990–2017. The longest black line shows expected values across the spectrum of the SDI. Shading indicates 95% confidence intervals

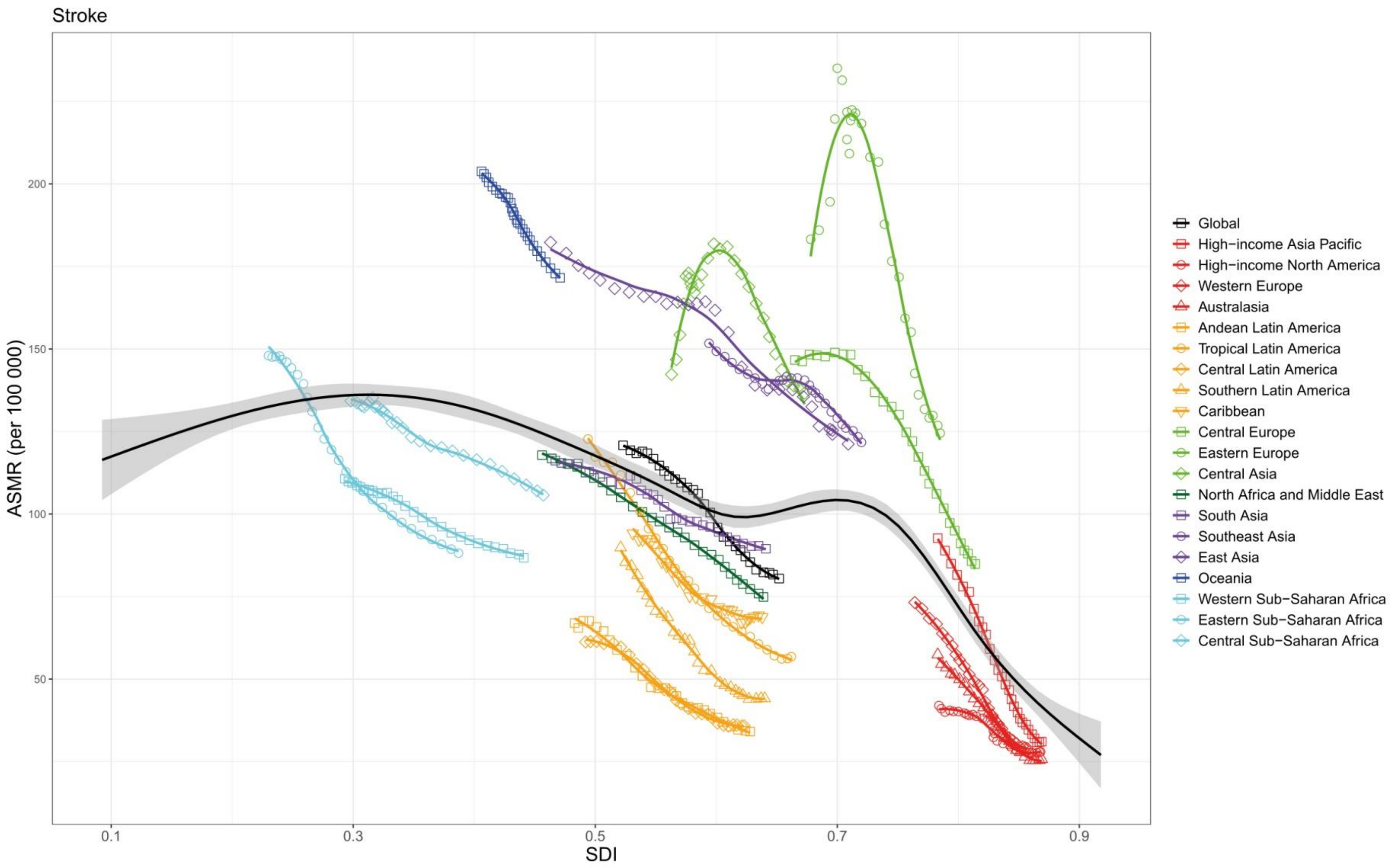

Supplementary fig 8 Age-standardized mortality rate of stroke for 21 GBD regions, 1990–2017. The longest black line shows expected values across the spectrum of the SDI. Shading indicates 95% confidence intervals

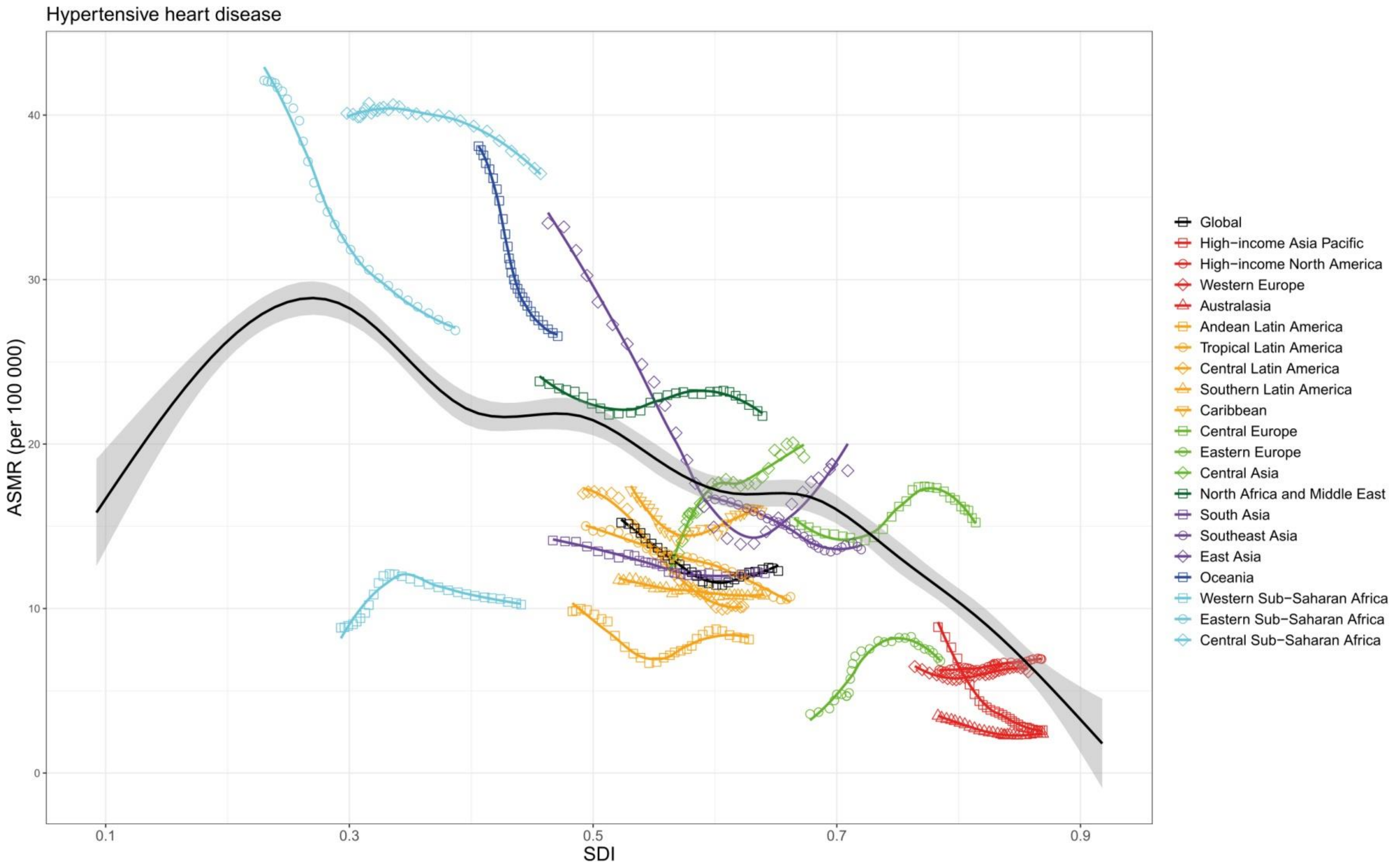

Supplementary fig 9 Age-standardized mortality rate of hypertensive heart disease for 21 GBD regions, 1990–2017. The longest black line shows expected values across the spectrum of the SDI. Shading indicates 95% confidence interva

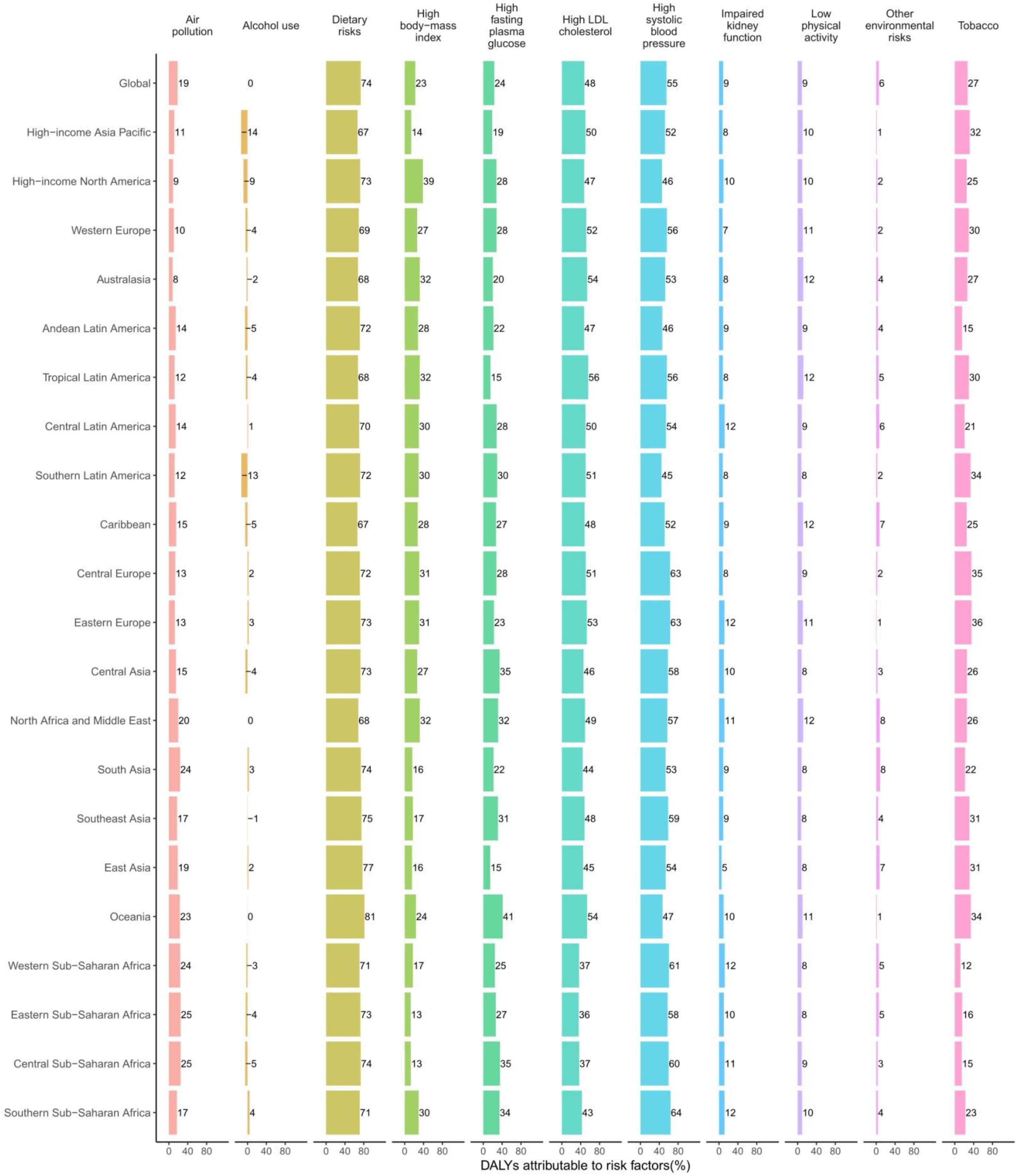

Supplementary fig 10 Percentage of age-standardized DALYs due to ischemic heart disease attributable to risk factors for 21 GBD regions, both sexes, 2017

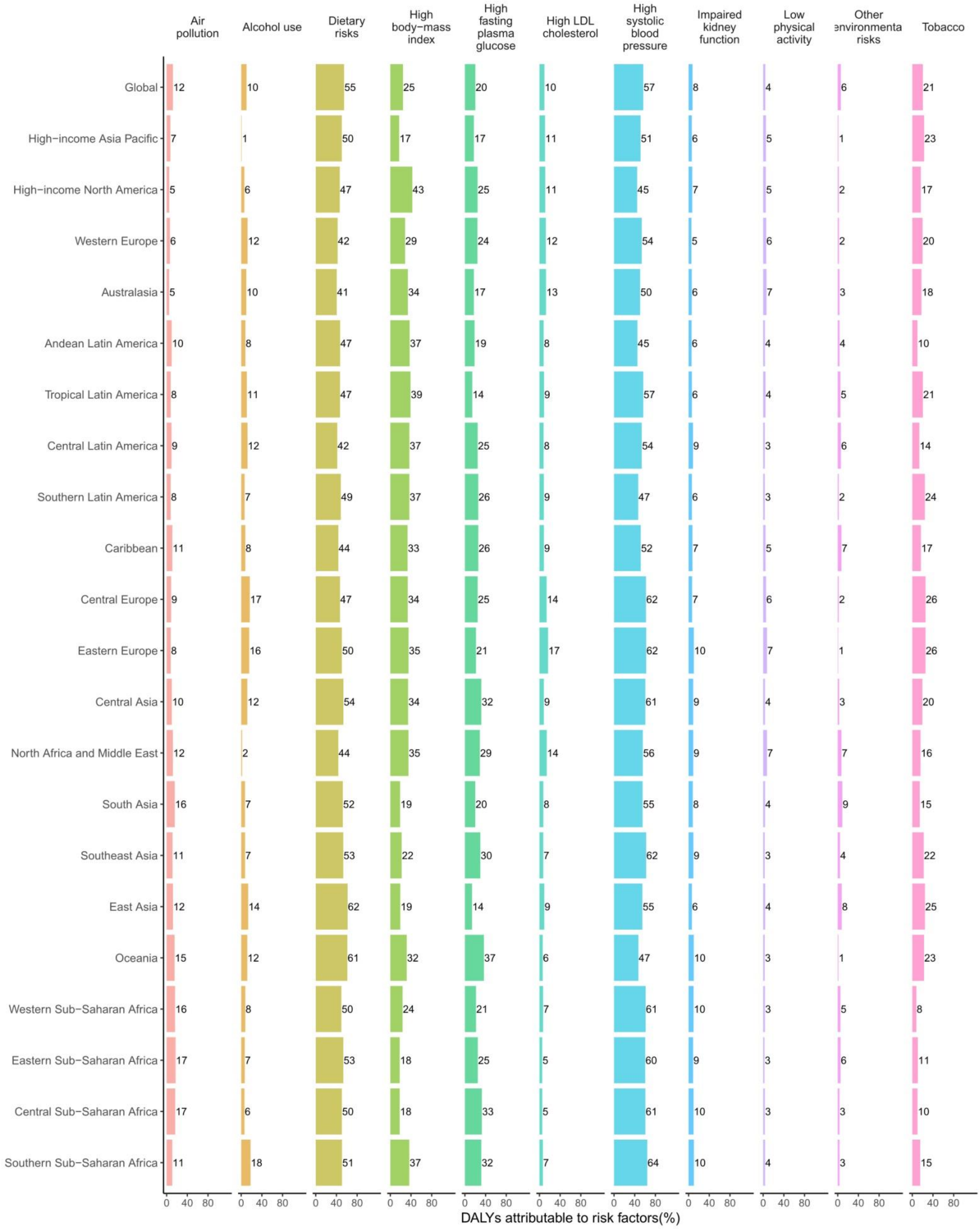

Supplementary fig 11 Percentage of age-standardized DALYs due to stroke attributable to risk factors for 21 GBD regions, both sexes, 2017
